# Specific design strategies for elderly-friendly residential spaces in nursing homes: an integrated model approach based on empathy map-KANO-AHP-PUGH

**DOI:** 10.3389/fpsyg.2026.1824564

**Published:** 2026-05-11

**Authors:** Zhu Jintao, Gao Weisong, Zhu Ning, Wang Lei

**Affiliations:** 1 Department of Design, School of Fine Arts and Design, Hebei Normal University, Shijiazhuang, China; 2 School of Architectural Art and Design, LuXun Academy of Fine Arts, Shenyang, China

**Keywords:** age-friendly environments, AHP, empathy maps, KANO model, PUGH, spatial design

## Abstract

**Objective:**

To address the challenges older adults face in resting within an aging society, this study explores the design characteristics of age-friendly residential spaces in nursing homes. The goal is to alleviate psychological stress stemming from rest difficulties and enhance the quality of life for seniors receiving home-based care.

**Method:**

First, explicit and implicit needs were identified through interviews combined with Empathy Maps. Next, the KANO model was applied to classify these needs and clarify design optimization directions. Subsequently, the Analytic Hierarchy Process (AHP) was used to construct an evaluation index system, determining the weighting and priority ranking of each need. Finally, the PUGH decision matrix was employed to compare and evaluate multiple design proposals, selecting the optimal solution. Applying research findings to the practical design of residential spaces in nursing homes.

**Results:**

Utilizing the Empathy Map, KANO model, AHP method, and PUGH decision matrix, an age-friendly residential spaces was designed to meet the physiological and psychological needs of the elderly.

**Conclusion:**

Integrating the KANO model with the AHP hierarchical analysis method enables precise identification of user needs while reducing arbitrary subjective assumptions. The PUGH decision matrix generates optimal design solutions to guide age-friendly residential space design. This study proposes a preliminary integrated decision-support framework that employs a structured process to conduct needs analysis and solution screening, which can serve as a reference for optimizing residential spaces for the elderly. Further validation is required through post-occupancy evaluations and user testing.

## 1 Introduction

Since the 21st century, the world's population has been aging at an accelerated pace. According to projections in the WHO's 2021 World Aging Report, the number of people aged 60 and over will rise from 730 million in 2020 to over 2 billion by 2050 (Steeper et al., 2016). The proportion of the global population aged 65 and older has risen from 6% in 1990 to 9% in 2019, and is projected to reach 16% by 2050 (Steeper et al., 2016). A previous study found that 28% of elderly individuals in China experience psychological loneliness (Luo and Waite, 2014). Due to the one-child policy, most couples have only one child, presenting elderly individuals with challenges and risks associated with loneliness. Retirement, living alone, and a lack of care from descendants exacerbate feelings of loneliness among the elderly (Hu and Wang, 2023; Jeste et al., 2020). This significant demographic shift toward an aging population presents multifaceted challenges, exacerbating systemic issues including the burden of chronic diseases, increasing demand for healthcare resources, and placing greater societal pressure on care systems (Khan et al., 2024).

Sleep disturbances are a key factor affecting health perception, with sleep patterns undergoing characteristic changes during the aging process (Wan et al., 2025). Research indicates that approximately one-third of adults experience sleep disorders on a daily basis (Ferrie et al., 2011). As age increases, sleep disturbances become increasingly prominent among the elderly population. Sleep disorders not only severely threaten the physical and mental health of older adults but have also emerged as one of the critical public health challenges facing aging societies (Zhong et al., 2019). Current research reveals that sleep disorders among the elderly may trigger various psychological disorders, cognitive impairments (Dzierzewski et al., 2018), and a decline in immune function (Piber et al., 2022). Consequently, traditional housing design models, particularly existing rest space designs, increasingly struggle to address the practical challenges faced by seniors regarding health, safety, emotional needs, and daily usability (Hoof et al., 2021). Compared to individual physical capabilities, the built environment exerts a more profound influence on the mental health of older adults (Zhou et al., 2022). A well-designed built environment has become crucial for enhancing older adults' residential satisfaction and subjective wellbeing (Tang et al., 2021). Providing adaptable residential spaces for the elderly is increasingly vital for ensuring their independent and healthy lifestyles (Diepstraten et al., 2020).

Current research has demonstrated that age-friendly environments positively correlate with both physical and mental health in older adults, with a more pronounced impact on mental health. The number of environmental factors moderates their association with physical health (Zhou et al., 2022). However, existing studies still exhibit three core limitations. ① Existing research on elderly housing needs predominantly focuses on explicit requirements like physical safety, while exploration of implicit needs such as psychological comfort and emotional belonging remains incomplete. Few studies employ tools like empathy maps for detailed needs analysis, making it difficult to fully capture genuine pain points and latent demands during elderly housing use. ②Current age-friendly sleeping space design predominantly relies on designers' experience to prioritize needs and select solutions (Li and Zhang, 2025). This approach fails to accurately categorize and weight needs, nor does it reduce subjective assumptions in design decision-making, resulting in designs that inadequately match the actual needs of older adults. ③While existing research confirms the positive impact of age-friendly environments on seniors' physical and mental health, it lacks effective pathways to translate multi-model integrated findings into practical design solutions. The absence of a closed-loop system spanning from needs analysis to implementation hinders the transition of age-friendly sleeping space design from experience-driven to data-driven approaches (Ji et al., 2023).

To address the limitations of previous studies, this research integrates multiple methodological approaches. Through semi-structured interviews with an independent sample, combined with the construction of empathy maps and KANO model analysis based on another independent questionnaire sample, we conduct an in-depth and objective exploration of the explicit and implicit needs of older adults and classify them accordingly. Utilizing the Analytic Hierarchy Process (AHP) and the PUGH decision matrix, it completes the design of age-friendly residential spaces that meet the physiological and psychological needs of the elderly. This approach paves new pathways for thinking and research methods in optimizing and enhancing age-friendly spaces. Establish a quantitative framework for analyzing requirements and selecting solutions for age-friendly sleeping quarters. Bridge the gap between theoretical models and practical design, driving a shift from experience-driven to data-driven design.

## 2 Materials and methods

### 2.1 Participants

To achieve the research objectives, we employed a purposive sampling method, selecting a total of 40 elderly individuals aged 60 and above (20 males and 20 females) from a community in Shijiazhuang. The target audience possesses basic language skills and can clearly articulate their residential experiences and requirements. The elderly sample was grouped according to MMSE cognitive assessment scores to facilitate differentiation of cognitive decline severity. All participants scored ≥21 on the MMSE and were able to articulate their subjective experiences fluently. All 40 participants underwent MMSE assessment and were categorized into two groups based on their scores (Normal: ≥26 points; Mild impairment: 21–25 points). Following stratification by MMSE scores (normal/mild impairment), randomization was conducted within each stratum to form a total of eight groups (*n* = 5 per group). The sample of the study population is shown in Table 1. This study was conducted in accordance with the Declaration of Helsinki and was approved by the Ethics Committee of Hebei Normal University. Prior to commencing the study, all participants and their family members (where cognitive impairment risk exists) were provided with a detailed explanation of the research objectives, content, and procedures. Participants voluntarily signed written informed consent forms, explicitly acknowledging their right to withdraw from the study at any time without providing reasons. Upon withdrawal, relevant data would be promptly destroyed. Interview questions avoided topics likely to cause psychological distress. Participants who mentioned negative experiences such as sleep disorders were offered necessary emotional support and guidance. Research findings will be communicated to participants in the form of a summary report, providing guidance for improving their home sleeping environment.

**Table 1:** Study population sample.

Age group	Stage characteristics	Behavior	Behavioral needs
60–70 younger elderly	Physical functions remain generally sound, often accompanied by mild chronic conditions (such as hypertension), with cognitive abilities largely intact.	Actively participate in social activities and interest-based courses (such as calligraphy and choir); proactively expand social circles in communal spaces; try balcony gardening and reading.	Primarily featuring single/twin standard rooms with private balconies/leisure corners; conveniently located near the communal lounge, activity rooms and outdoor garden.
71–80 middle-aged and elderly persons	High prevalence of chronic conditions (diabetes, arthritis, etc.), mild cognitive decline, with some individuals experiencing hearing/vision impairment.	Prefers sedentary leisure activities (board games, television viewing); Frequently visits the nursing station to consult on health matters; Relies on children or carers for companionship in communal areas.	Primarily featuring standard rooms with age-friendly modifications, including grab rails and non-slip flooring in bathrooms; quiet rest areas provided in communal spaces.
81 and above senior citizens	Significant decline in physical function, high risk of disability/dementia, often accompanied by multiple severe chronic conditions.	Mobility dependent on wheelchair/bed; individuals with cognitive decline are prone to memory confusion and emotional fluctuations; they form strong attachments to familiar carers, with family members frequently visiting at bedside.	Primarily comprising nursing-style single and twin rooms, each equipped with bedside call systems and bathing assistance facilities; situated adjacent to the nurses' station and rehabilitation room; public areas feature designated bedside activity zones.

### 2.2 Site selection for research

This study selected the Chang'an Community Senior Apartments in Shijiazhuang, the provincial capital of Hebei and a central city within the Beijing-Tianjin-Hebei region, as its research site. From three dimensions—demographic structure, economic foundation, and policy orientation—this location offers distinct advantages for conducting research on age-friendly residential spaces. The specific analysis is as follows:

## Demographic dimension: pronounced aging characteristics with strong sample representativeness.

As the provincial capital of Hebei, Shijiazhuang exhibits a progressively deepening trend of population aging. By the end of 2024, the proportion of residents aged 60 and above exceeded 19% citywide. Within the main urban district of Chang'an, the aging rate reached 22%, surpassing the municipal average. Existing care homes, predominantly constructed around the year 2000 with traditional layouts, present an urgent need for age-friendly retrofitting. This situation provides researchers with a typical and rich sample dataset for analysis.

## Economic dimension: robust economic strength and comprehensive supporting facilities.

Shijiazhuang City exhibits sound economic development, providing a solid financial foundation for establishing elderly care service systems and implementing age-friendly renovations. The community enjoys convenient transport links and is surrounded by extensive commercial and medical facilities, ensuring efficient progress of research activities while offering an excellent practical environment for implementing research findings (Wang Y. et al., 2025).

## Policy dimension: substantial policy support with solid practical foundation.

Shijiazhuang City places high priority on addressing aging-related challenges, incorporating age-friendly renovations into key tasks of urban renewal and livelihood projects. Policy documents such as the Shijiazhuang City 14th 5-Year Plan for Elderly Care Service System Development and the Implementation Plan for Age-Friendly Renovations in Urban Old Residential Areas have been successively issued. These explicitly advocate optimizing residential environments for the elderly and promoting standardized construction of home-based elderly care facilities. Community residents demonstrate high acceptance and willingness to participate in age-friendly renovations. Preliminary small-scale modifications, such as installing handrails and applying anti-slip flooring, have already been implemented, establishing a solid practical foundation for the successful execution of this research (Xu et al., 2024). In summary, the Chang'an Community Senior Apartments in Shijiazhuang provide an ideal research vehicle for studying age-friendly residential spaces across three dimensions: demographic composition, economic infrastructure, and policy support. The selection of this site aligns with core global trends in aging research while ensuring feasibility through local practical foundations. It enables the identification of core pain points in recreational accessibility through a high proportion of elderly residents, and leverages policy and facility advantages to facilitate the practical application of research findings. This selection establishes a robust foundation for the subsequent application of the integrated KANO-AHP-PUGH multi-model methodology, while also providing a representative case study supporting the transition of age-friendly residential space design from experience-driven to data-driven approaches.

### 2.3 Research methods

Within the field of design research, scientific design methodologies serve to enhance design efficiency. Empathy maps constitute a user-centered research approach designed to assist teams in gaining a profound understanding of users' emotions, thoughts, motivations, and behaviors (Ölcer et al., 2025). Empathy maps are widely employed during the design research phase. By visualizing users' thoughts and feelings, they assist researchers in identifying user needs and pain points, thereby enabling better decision-making. Typically comprising four quadrants, they document respondents' verbal expressions, emotional experiences, cognitive processes, and behavioral manifestations, thereby facilitating in-depth exploration and understanding of interviewees within the team (Chen and Tsai, 2024).

**Figure 1:**
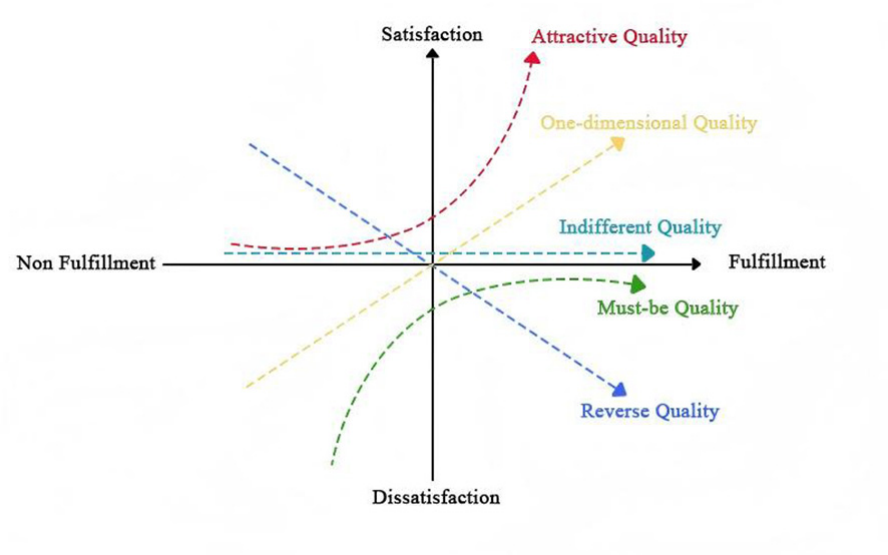
Relationships between elements of the KANO model.

The Kano model is a user requirement analysis framework proposed by Dr Noriaki Kano, Professor at Tokyo Polytechnic University, Japan, during the 1970s. It addresses issues of requirement matching and prioritization by distinguishing relationships between different demands (Yadav et al., 2013). This model effectively reflects the relationship between product or service quality and user satisfaction. The Kano model categorizes user requirements into five distinct groups, each representing a different level of user expectation toward products and services (Zhang et al., 2024). The relationships between the elements of the KANO model are illustrated in Figure 1 (Qian et al., 2025).

Must-have quality: also referred to as fundamental requirements, these represent users' core, foundational expectations of products and services, constituting the baseline demands that must be met. Users implicitly assume such requirements ‘should exist by default'; fulfilling them does not significantly enhance satisfaction, yet failure to do so will provoke extreme dissatisfaction.

One-dimensional quality: this refers to explicit requirements clearly articulated by users, where the degree of fulfillment exhibits a linear positive correlation with user satisfaction: the higher the level of requirement fulfillment, the greater the user satisfaction; conversely, lower fulfillment leads to diminished satisfaction.

Attractive quality: also known as the “excitement-type” requirement, this refers to implicit user needs that are not explicitly articulated. Users typically do not actively expect such requirements to be fulfilled, yet their satisfaction delivers unexpected delight, significantly enhancing overall satisfaction. Even when unmet, users do not experience dissatisfaction.

Indifferent quality: the fulfillment of such requirements has no discernible impact on user satisfaction. Users neither experience heightened satisfaction when these needs are met nor feel dissatisfied when they remain unmet. These typically constitute non-core, non-essential supplementary requirements.

Reverse quality: the degree to which such requirements are met exhibits an inverse relationship with user satisfaction: the more fully the requirements are satisfied, the lower the user satisfaction becomes. These requirements typically conflict with users' usage habits and core demands, with some users even developing resistance due to their very existence.

The five categories within the KANO model provide a comprehensive classification system for understanding user requirements. Analyzing user needs through this framework enables a clearer grasp of requirement attributes, establishes prioritization for product design and development, and assists developers in navigating the design trade-off process (Wang T. et al., 2025).

The Analytic Hierarchy Process (AHP) is a systematic analytical method combining qualitative and quantitative analysis, proposed by Professor Laffey Saaty, an American operations researcher, in the early 1970s. The AHP is a subjective weighting method that calculates scores for individual indicators through expert ratings, thereby deriving a judgement matrix for pairwise comparisons between indicators (Whitaker, 1987). The Analytic Hierarchy Process (AHP) is widely applied in design, engineering, and environmental management, regarded as a reliable tool for complex decision-making tasks. Within the cultural and creative products sector, AHP facilitates the assessment of the relative importance of various design factors (Wang Z. et al., 2025). Its advantage lies in enabling hierarchical weighting analysis of user requirements, assisting designers in comprehensively evaluating the relative significance of different user needs and guiding subsequent design iterations.

The PUGH decision matrix, first proposed by American management scholar Edward Pugh in 1960, is a decision-making model for evaluating and comparing multiple options, also known as the concept selection matrix (Zhu et al., 2022). The PUGH matrix can be employed to conduct preliminary screening and comprehensive evaluation of design proposals, identifying the optimal solution for implementation in the final design. This assists teams in overcoming subjective biases and making data-driven decisions. The PUGH decision matrix is widely adopted due to its simplicity, practicality, and logical rigor. It offers significant advantages during the later stages of design, particularly in functional selection and design optimisation (Violante and Vezzetti, 2017). This methodology substantially mitigates the risk of one-sided decisions based on singular criteria. Nevertheless, scoring and weighting allocation retain subjective elements, necessitating team consensus and professional judgement to prevent benchmark or standard-setting biases from influencing outcomes, thereby ensuring sound and reliable decision-making.

The KANO-AHP model proves effective in the user requirement discovery phase, yet it cannot evaluate subsequent specific design proposals during the design stage. The PUGH matrix compares alternative proposals against a reference solution to identify the optimal option. Integrating the KANO-AHP model with the PUGH matrix addresses the limitations inherent in employing either model independently. This combined approach enables a more precise and comprehensive assessment of user requirements, thereby guiding subsequent decision-making and enhancing the scientific rigor and accuracy of the design process.

In existing literature, the application of KANO, AHP and PUGH has been extensively explored across multiple domains. Fang et al. employed an integrated KANO-AHP-QFD-PUGH model to design a medication reminder application for the elderly, demonstrating that this methodology not only optimizes user experience but also fosters design innovation and sustainability (Fang et al., 2023). Dong et al. employed a combined KANO-AHP-QFD approach to design smart rooms for dementia care facilities (Dong and Zhu, 2026). Wang et al. designed a walking aid for the elderly based on the KANO-AHP-FEC methodology, applying the integrated model to age-friendly design (Wang T. et al., 2025). While previous studies have applied multi-model integration to age-friendly design, this study is the first to deeply integrate the Empathy Map with the KANO-AHP-PUGH model and apply it to the physical design of residential spaces in senior care facilities. In addition, Zhu et al. introduced the combined use of AHP and PUGH decision matrices into the innovative design of surgical assistive devices, effectively mitigating subjective factors in product design and enhancing its scientific rigor (Zhu et al., 2022). Bao Qian et al. combined the GT-KANO-AHP-QFD integrated method with user-centered design to study parent-child interactive seating in urban children's parks (Bao et al., 2025). Although existing research has extensively explored the integrated application of Kano, AHP, and PUGH methodologies, a unified research paradigm remains unestablished. The application of these methods often necessitates contextual customization.

**Figure 2:**
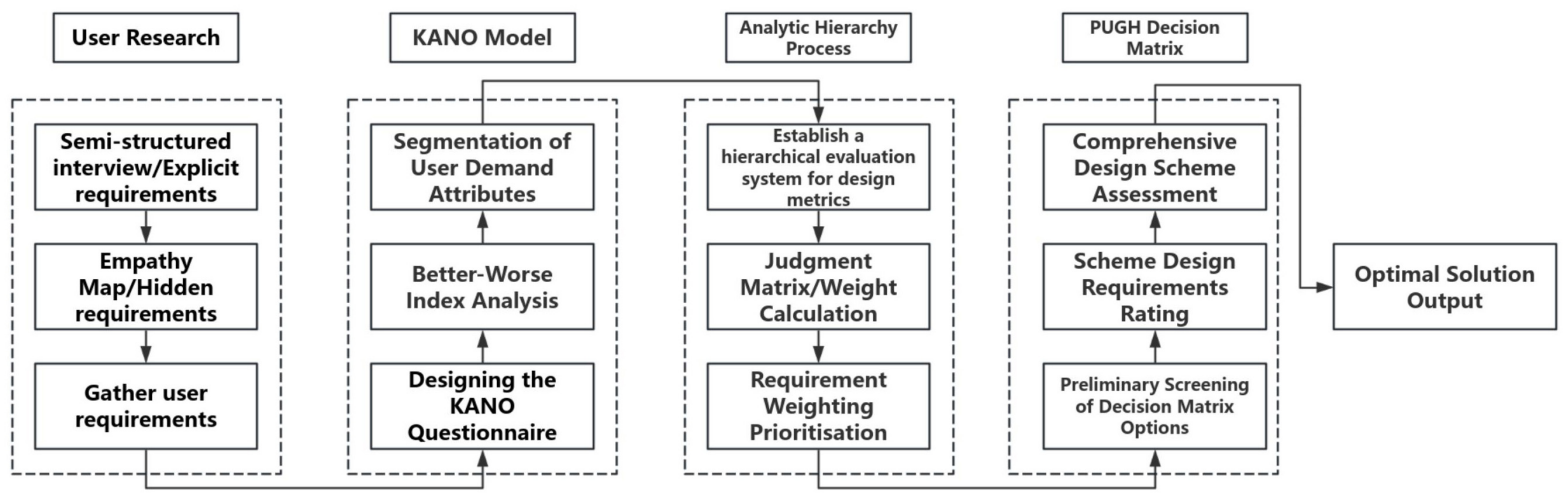
Research process for the combined design of empathy map-KANO-AHP-PUGH

This study employs empathy mapping, the KANO model, AHP, PUGH, and continuous integration methods. Rather than simply combining established tools, it represents a systematic integration designed to address pain points throughout the entire process of designing age-friendly residential spaces. This paper innovatively incorporates the construction of an Empathy Map into preliminary interviews with elderly users, thereby identifying latent needs and design pain points among the elderly. It combines the KANO model with AHP analysis: the KANO model categorizes user requirements (Neira-Rodado et al., 2020), while AHP hierarchical analysis determines the relative importance of each requirement, establishing a weighted ranking among them. The integrated Empathy Map-KANO-AHP-PUGH model is applied to design age-friendly residential spaces, thereby validating the methodology's applicability within this field. The research process for the Empathy Map-KANO-AHP-PUGH framework is illustrated in Figure 2.

## 3 Result

### 3.1 Building empathy maps

To achieve the research objectives, we selected 40 elderly individuals capable of independent living as interview subjects. During the interviews, the research team prioritized establishing positive rapport with participants, deliberately conducting interviews in quiet and comfortable settings. A semi-structured format was employed, with follow-up inquiries made regarding interviewees' responses. This approach enables more direct and effective elicitation of user needs (Fu et al., 2023). The interview outline was designed around three key dimensions: “spatial layout adaptability,” “functional facility adaptability,” and “environmental atmosphere adaptability.” Specific interview questions are detailed in the Appendix Table.

**Figure 3:**
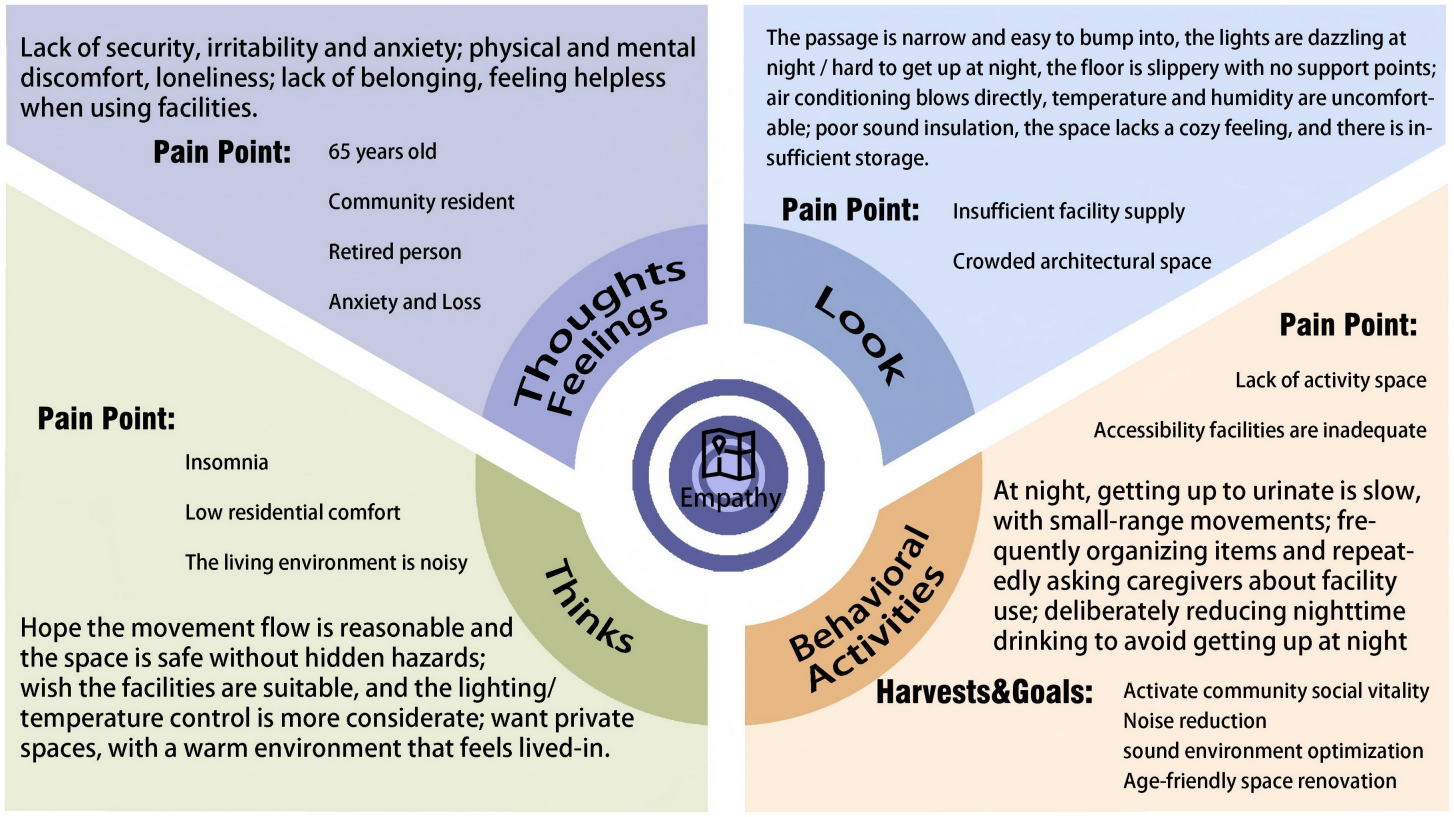
Empathy map

Following our interviews with elderly individuals, we employed the Empathy Map methodology to construct a map based on the interview content. This approach enabled us to empathize with and systematically organize the implicit needs of the elderly. The Empathy Map primarily consists of elements such as feelings, visual perceptions, and auditory experiences. The Empathy Map is shown in Figure 3.

Based on actual interview dialogues with seniors and the construction of empathy maps, we identified their practical needs for sleeping and residential spaces. First, by analyzing seniors' complaints about safety hazards in interviews and the safety concerns reflected in the “perceived behavior” dimension of the empathy map, we derived Safety Adaptation Requirement A, which aligns with aging-in-place needs. Second, by focusing on the inconvenience elderly individuals experience when using facilities, we derive the “functional mismatch” resulting from their declining behavioral capabilities. This leads us to functional practical needs *B* that align with the physiological characteristics of the elderly. Finally, by deeply exploring the psychological needs of the elderly through empathy maps and uncovering the emotional expressions implicit in interviews, and by integrating mainstream perspectives from existing age-friendly research, designs that align with the psychological needs of older adults can better accommodate their living conditions. This approach fulfills their emotional comfort needs—specifically, the need for a sense of belonging and self-respect. We have compiled safety adaptation needs, functional utility needs, and emotional comfort needs into a table, yielding preliminary requirements for the sleeping and residential spaces of the elderly. Preliminary Requirements for Elderly Residential Spaces are shown in Table 2.

**Table 2:** Functional requirements analysis table.

Demand dimension	Number	Specific requirements	Function
Security adaptation requirements	Q1	No elevation difference in the flow path	No elevation changes on the floor. Passage width ≥ 1.2 m. Corner turning space reserved ≥ 1.5 × 1.5 m.
	Q2	Handrail anti-slip load-bearing	Install additional non-slip grab bars near the bed and in the bathroom. Rated load capacity ≥120 kg. Height: 75–85 cm. Diameter: 30–40 mm. Securely mounted with smooth, rounded ends.
	Q3	Floor slip resistance	Use wear-resistant floor tiles with a slip resistance coefficient ≥0.6, featuring a non-reflective surface and easy-to-clean properties.
	Q4	Emergency call	Bedside and bathroom one-touch call buttons are installed at a height of 70–80 cm for easy operation. Response time is ≤ 3 min, with fault alerts and backup power supply.
	Q5	Collision-proof design	Furniture, wall corners, and door/window frames shall feature rounded edges (radius ≥ 10 mm), with no sharp edges or corners, and edges shall be sanded smooth.
	Q6	Light sensor	Install motion-sensing night lights in hallways, beside beds, and in bathrooms. Sensing range: 0.5–3 meters Brightness: 5–10 lux Automatically turns off 30 seconds after movement ceases
	Q7	Power supply electric shock protection	Outlets feature child-proof covers, mounted at 70–120 cm height, rated for 220 V, suitable for everyday and medical appliances.
Functional requirements	Q8	Multi-level lighting adjustment	Dual lighting with main lamp and bedside lamp, featuring independent brightness adjustment and soft on/off controls.
	Q9	Minimum focus distance	Storage cabinets are 70–150 cm tall and 40–50 cm deep. Nightstands are flush with the bed height and positioned ≤ 0.5 *m* from the bed.
	Q10	One-touch operation for appliances	One-touch operation for appliances Air conditioners, humidifiers, and more are controlled with a single button. The buttons are clearly marked, feature a timer function, and are easy to operate.
	Q11	Adjustable bedding	Low-age beds feature adjustable angles, while mid-to-high-age and elderly patients use lift-adjustable nursing beds with adjustable head and foot sections.
	Q12	Effortless curtain operation	Pull-and-slide/roller-style curtains with pull cords ≥1.2 m (non-slip grip), allowing light to pass through while blocking visibility, with easy adjustment.
	Q13	Temperature and humidity control	Set air conditioner temperature between 18–26 °C, with smart humidity control (40%−60%), featuring temperature and humidity display.
Emotional Comfort Needs	Q14	Privacy Partition Adjustment	The twin-bed accommodation features movable soft partitions that can be easily opened or closed, ensuring personal privacy while facilitating interaction between roommates.
	Q15	Personalized Space	Supports displaying personal photos, keepsakes, reading glasses, and other personal items to preserve traces of life.
	Q16	Natural lighting adapts to leisure	Equipped with lounge chairs and adjustable sunshades to ensure natural light exposure, perfect for sunbathing and reading.
	Q17	Fresh and pleasant scent	Use mild, odorless cleaning and disinfecting products. Place lavender or other natural aromatherapy items.
	Q18	Flexible Companion Space	Residences for elderly residents include designated family visitation areas; Residences for younger and middle-aged residents feature compact relaxation corners to facilitate visits and conversations with friends and relatives.
	Q19	Warm and consistent color scheme	Featuring soft, warm tones like off-white, the walls are finished in a matte texture, with furniture colors harmonizing with the walls.
	Q20	Green Plant Compatibility	Place non-toxic, thornless, low-maintenance green plants near windows and on bedside tables. Choose lightweight, non-slip pots to support easy care by seniors.
	Q21	Integration of nostalgic elements	A small photo display panel is reserved on the wall for attaching vintage photographs; optional retro-style decorative accessories are available.
	Q22	Creating a serene atmosphere	Features soft background music with an on/off switch, primarily featuring soothing melodies.

### 3.2 User needs and classification in the KANO model

Based on the aforementioned 22 preliminary age-friendly requirements, a KANO questionnaire was designed. Each requirement features two opposing questions: “Strongly agree,” “Somewhat agree,” “Neutral,” “Somewhat disagree,” and “Strongly disagree.” The KANO Model questionnaire design (partial version) is shown in Table 3. The complete version of the KANO Model questionnaire is included in the Appendix. The questionnaire was distributed from September to December 2025. This survey was conducted through in-person distribution. We randomly selected 120 elderly individuals aged 60 and older who reside in nursing homes as the subjects of our study. The participants in this survey and the interview subjects constitute two entirely separate research samples, but they were selected based on identical criteria (aged 60 or older, MMSE cognitive score of 21 or higher, capable of clearly expressing themselves and communicating their needs, and able to independently describe their living experiences). Invalid responses in this survey are determined based on the following three criteria; a survey is deemed invalid if it meets any one of them: ① The survey is incomplete, with more than three core questions (Q1–Q22) left unanswered; ② The survey contains obvious signs of careless completion, such as selecting the same scale option for all questions, or obvious logical contradictions between responses to positive and negative questions (e.g., indicating both “strongly agree to provide” and “strongly agree not to provide” for the same need); ③ The respondent does not meet the study requirements; the questionnaire was not completed by a resident aged 60 or older in a long-term care facility. A total of 120 questionnaires were distributed, with 120 returned and 6 deemed invalid, resulting in a 95% response rate. Based on the survey findings and using the KANO evaluation framework as a reference, demand attributes were categorized. KANO evaluation results classification comparison table in Table 4. The summary of design requirements for residential spaces in senior apartments is presented in Table 5.

**Table 3:** KANO model questionnaire design (partial version).

Number	Problem	Strongly agree	Somewhat agree	Neutral	Somewhat disagree	Strongly disagree
Q1	What is your stance on elevation differences in floor plans?					
	What is your stance on elevation differences in floor plans?					
Q2	What is your stance on the anti-slip and load-bearing properties of handrails?					
	What is your stance on non-handrail, slip-resistant load-bearing structures?					

**Table 4:** KANO evaluation results classification comparison table.

User requirements		The sleeping quarters do not have this function				
The sleeping quarters are equipped with this feature.		Strongly agree	Somewhat agree	Neutral	Somewhat disagree	Strongly disagree
	Strongly agree		A	A	A	O
	Somewhat agree	R	I	I	I	*M*
	Neutral	R	I	I	I	*M*
	Somewhat disagree	R	I	I	I	*M*
	Strongly disagree	R	R	R	R	

**Table 5:** Summary of design requirements for residential spaces in senior apartments.

Serial number	A	O	*M*	I	R	Causality
Q1	19	66	11	15	3	O
Q2	11	77	9	9	8	O
Q3	8	51	21	24	10	O
Q4	12	7	80	14	1	*M*
Q5	14	13	75	10	2	*M*
Q6	12	13	70	14	5	*M*
Q7	74	13	12	12	3	A
Q8	19	65	21	5	4	O
Q9	21	59	11	10	13	O
Q10	6	22	15	63	8	I
Q11	13	68	13	15	5	O
Q12	61	13	9	20	11	A
Q13	69	14	13	16	2	A
Q14	12	70	12	15	5	O
Q15	55	21	17	13	8	A
Q16	60	14	17	13	10	A
Q17	59	23	13	14	5	A
Q18	12	24	13	60	5	I
Q19	12	63	12	22	5	O
Q20	15	64	15	11	9	O
Q21	6	18	21	23	46	R
Q22	15	12	62	15	10	*M*

Based on detailed data collected from user needs surveys, the Satisfaction Index (*Is*) and Dissatisfaction Index (*Ids*) for functional requirements can be calculated. Typically, IS is positive while IDS is negative, as shown in Equations (1) and (2).


$$\begin{array}{l} SatisfactionIndex:=\;\frac{A\;+\;O}{A\;+\;O\;+\;M\;+\;I} \end{array}$$ (1)

$$\begin{array}{l} DissatisfactionIndex:=\left(-1\right)\times \;\frac{O\;+\;M}{A\;+\;O\;+\;M\;+\;I} \end{array}$$ (2)

**Figure 4:**
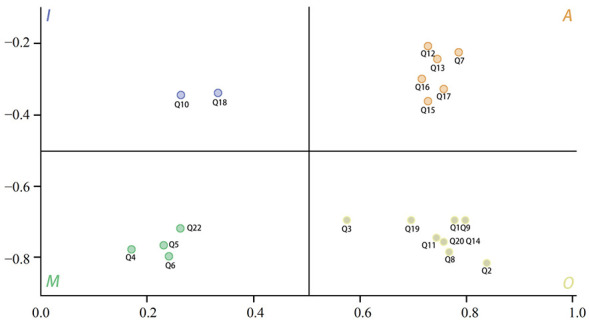
Four-quadrant classification diagram.

In (1), Is represents the Better coefficient, indicating the user's satisfaction coefficient for the i-th feature. In (2), Ids represents the Worse coefficient, indicating the user's dissatisfaction coefficient for the same feature. Ai, Oi, Mi, and Ii respectively denote the percentage of users selecting the four demand types A (Attractive), O (One-dimensional), *M* (Must-be), and I (Indifferent) for each feature in the questionnaire survey. Using the above equations, the values for the 22 design requirements were substituted into the calculations, yielding the results shown in Table 6. To more clearly illustrate the importance of residential space requirements in nursing homes for elderly users, we employ a quadrant diagram to visualize the Better-Worse Index for each metric (Li et al., 2023). The quadrant classification based on data functional attributes is shown in Figure 4.

**Table 6:** Analysis results of better-worse indicators for various demands.

Serial number	Is	Ids	Serial number	Is	Ids
Q1	0.77	−0.69	Q12	0.72	−0.21
Q2	0.83	−0.81	Q13	0.74	−0.24
Q3	0.57	−0.69	Q14	0.75	−0.75
Q4	0.17	−0.77	Q15	0.72	−0.36
Q5	0.24	−0.79	Q16	0.71	−0.30
Q6	0.23	−0.76	Q17	0.75	−0.33
Q7	0.78	−0.23	Q18	0.33	−0.34
Q8	0.76	−0.78	Q19	0.69	−0.69
Q9	0.79	−0.69	Q20	0.75	−0.75
Q10	0.26	−0.35	Q21	0.35	−0.57
Q11	0.74	−0.74	Q22	0.26	−0.71

### 3.3 User requirement ranking using the AHP method

**Figure 5:**
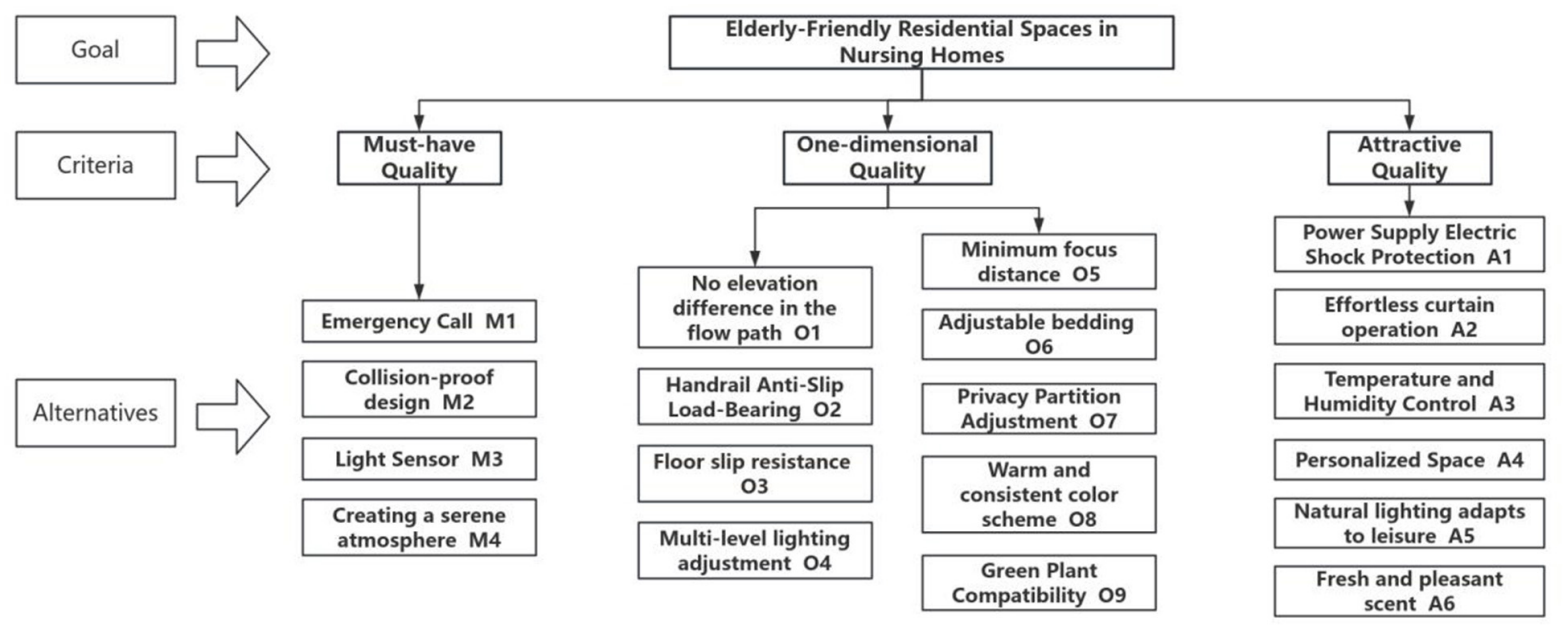
AHP hierarchical structure.

Based on the analysis results of the KANO questionnaire and in conjunction with the core requirements for designing residential spaces in nursing homes, a three-tier hierarchical model comprising the objective layer, criterion layer, and alternatives layer was constructed. This model clearly defines the analytical indicators for each level, laying the foundation for subsequent construction of the judgment matrix and weight calculations. The hierarchical structure is illustrated in Figure 5.

1) Objective layer: the objective layer in this study is Design *B*, which achieves an age-friendly residential space for nursing homes.

2) Criteria layer: the criteria layer in this study comprises Essential Requirements (*M*), Desired Requirements (O), and Attractive Requirements (A).

3) Alternatives layer: the sub-criteria layer in this study is divided into 19 items. Based on the results of the KANO model-based needs classification, we conducted a compliance screening of the 22 preliminary needs identified for nursing home residential spaces. We eliminated reverse-type (R) and irrelevant (I) needs, ultimately selecting 19 valid needs to form the candidate set, ensuring that all evaluation indicators represent needs with positive design value for nursing home residential spaces. We formed an expert panel consisting of 15 university professors and associate professors specializing in design to conduct the AHP scoring.

Since the relative importance of various criteria is difficult to quantify directly, the Analytic Hierarchy Process (AHP) constructs a judgment matrix using pairwise comparisons. This process employs a total of 17 values—ranging from 1 to 9 and their reciprocals—as a rating scale to determine the values of the matrix elements, known as the commonly used 9-point scale. The specific meanings corresponding to each rating level are shown in Table 7.

**Table 7:** Judgment matrix scaling.

Scale	Meaning of the scale	Ratio
1	When comparing element i in the former list with element j in the latter, i and j are equally important	a_ij_ = 1
2	When comparing element i in the former set with element j in the latter, i is slightly more important than j	a_ij_ = 3
3	When comparing element i in the former set with element j in the latter, i is clearly more important than j	a_ij_ = 5
4	When comparing element i in the former set with element j in the latter set, i is significantly more important than j	a_ij_ = 7
5	When comparing element i in the former set with element j in the latter set, i is absolutely more important than j	a_ij_ = 9
6	indicates that the importance of element i and element j lies between the two values mentioned above	a_ij_ = 2, 4, 6, 8
7	If the relative importance scale between element i and element j is a_(ij), then the relative importance scale between element j and i is aji = 1/aij	countdown

Suppose the *n* factors associated with the higher-order factor *z* are x_1_, x_2_, …, x_n_, where *i, j* = 1, 2, …, *n*, and let a_ij_ denote the importance ratio of factor x_i_ relative to factor x_j_ with respect to *z*. Based on these ratios, a complete pairwise comparison matrix A for these *n* factors with respect to the higher-order factor *z* can be scientifically constructed, with the matrix structure shown in (3).


$$\begin{array}{l} A_{n\;\times \;n}\;=\;\left[\begin{array}{cccc} a_{11} & a_{12} & a_{1..} & a_{1n}\\ a_{21} & a_{22} & a_{2..} & a_{2n}\\ a_{..} & a_{..} & a_{..} & a_{..}\\ a_{n1} & a_{n2} & a_{n..} & a_{nn} \end{array}\right] \end{array}$$ (3)

By integrating the professional judgements of multiple experts, we employ the geometric mean method to scientifically combine the judgement matrices constructed by each expert. This method effectively ensures the reliability of the consistency test for the final integrated matrix. The specific procedure involves taking the individual expert scoring matrices formed by the *m* experts participating in the evaluation (*m* = 1, 2, ..., k), multiplying the corresponding elements in each row and column, and then taking the mth root of the resulting product to obtain a unique comprehensive integrated judgement matrix. The specific mathematical formula is expressed as in (4).


$$\begin{array}{l} \bar{A}\;=\;{\left(\prod_{k\;=\;1}^{m}a_{ij}^{k}\right)}^{\frac{1}{m}} \end{array}$$ (4)

Based on the fundamentals of matrix theory, we employ the geometric mean method (also known as the square root method) to calculate the weights of the single decision matrix that has been established through expert consensus. This method has been proven in practice to offer a high degree of reliability and computational efficiency. The specific mathematical formula is given in (5).


$$W_{i}\;=\;\frac{(\prod_{j\;=\;1}^{n}a_{ij}{)}^{\frac{1}{n}}}{\sum_{i\;=\;1}^{n}(\prod_{j\;=\;1}^{n}a_{ij}{)}^{\frac{1}{n}}}\;,\;i\;=\;1,\;2,\;3..,\;n$$ (5)

In the practical process of constructing a judgement matrix, due to the inherent complexity of real-world problems and the inherent diversity of human cognition, experts find it difficult to specify an absolutely precise ratio between two factors; they can only make reasonable estimates based on experience. This inevitably leads to a certain degree of deviation between the values in the judgement matrix and the actual ratios that would exist under ideal conditions; consequently, it is virtually impossible to ensure that the judgement matrix is entirely consistent.

The consistency ratio (*CR*) is widely adopted in academia as an objective standard for assessing the consistency of a judgment matrix; the *CR* is defined as the ratio of the consistency index (CI) to the random consistency index (*RI*) of the same order. When the calculated *CR* value is less than 0.1, this indicates that the decision matrix possesses an acceptable level of consistency and requires no adjustment; conversely, a team of experts must review and revise the original decision matrix until the final calculation meets the scientific requirement of *CR* < 0.1. The calculation formulas are given in Equations (6) and (7).


$$\begin{array}{l} CI\;=\;\frac{\lambda_{\max}-n}{\left(n-1\right)} \end{array}$$ (6)

$$\begin{array}{l} CR\;=\;\frac{CI}{RI}\;=\;\frac{\lambda_{\max}-n}{\left(n-1\right)RI}<0.1 \end{array}$$ (7)

The maximum eigenvalue is denoted by λ_max_, and the decision matrix under examination is denoted by *n*. The calculation formula is shown in Equation (8). The average random consistency index is denoted by *RI*; it is positively correlated with the order of the decision matrix, as shown in Table 8.


$$\begin{array}{l} \lambda_{\max}\;=\;\sum_{i\;=\;1}^{n}\frac{{\left[\bar{A}W\right]}_{i}}{nW_{i}} \end{array}$$ (8)

**Table 8:** *RI* values for different orders.

Matrix order	1	2	3	4	5	6	7	8	9	10	11	12
*RI*	0	0	0.52	0.89	1.12	1.26	1.36	1.41	1.46	1.49	1.52	1.54

We first calculated the weights for the target-level indicators: must-have Quality (*M*), One-dimensional Quality (O) and Attractive Quality (A). After calculating and ensuring that the judgment matrices constructed by the 15 experts all met the consistency test criteria (*CR* < 0.1), we performed matrix aggregation on the 15 judgment matrices using Formula (4). The matrix obtained following aggregation is shown in Table 9A.

**Table 9:** Criteria layer judgment matrix and weights.

(A)			
Requirement type	Must-have quality (*M*)	One-dimensional quality (O)	Attractive quality (A)
Must-have quality (*M*)	1	2.6977	5.0718
One-dimensional quality (O)	0.3707	1	2.5487
Attractive quality (A)	0.1972	0.3924	1
(B)							
Requirement type	Must-have quality (*M*)	One-dimensional quality (O)	Attractive quality (A)	Weight	λ_max_	*CI*	*CR*
Must-have quality (*M*)	1	2.6977	5.0718	0.6289			
One-dimensional quality (O)	0.3707	1	2.5487	0.2621	3.0104	0.0052	0.0100
Attractive quality (A)	0.1972	0.3924	1	0.1091			

Based on Equations 5, 6, 7, and 8, for the integrated matrix assessing Must-have Quality (*M*), One-dimensional Quality (O) and Attractive Quality (A), the weighting and consistency calculations are carried out as follows:

① Based on the table above, the decision matrix is obtained as follows:


$$\begin{array}{c} A\;=\;\left[\begin{array}{ccc} 1.0000 & 2.6977 & 5.0718\\ 0.3707 & 1.0000 & 2.5487\\ 0.1972 & 0.3924 & 1.0000 \end{array}\right] \end{array}$$

② Multiplying the elements of $\bar{\text{A}}$ row by row yields a new vector *B*.


$$\begin{array}{c} B\;=\;\left[\begin{array}{c} 13.6822\\ 0.9448\\ 0.0774 \end{array}\right] \end{array}$$

③ Raise each component of the new vector *B* to the third power to obtain the eigenvector *M*.


$$\begin{array}{c} M\;=\;\left[\begin{array}{c} 2.3918\\ 0.9813\\ 0.4261 \end{array}\right] \end{array}$$

④ Normalizing the resulting vector *M* yields the weight vector *W*.


$$\begin{array}{c} W\;=\;\left[\begin{array}{c} 0.6296\\ 0.2583\\ 0.1122 \end{array}\right] \end{array}$$

⑤ Calculate the maximum eigenvalue λ_max_.


$$\begin{array}{c} AW\;=\;\left[\begin{array}{ccc} 1.0000 & 2.6977 & 5.0718\\ 0.3707 & 1.0000 & 2.5487\\ 0.1972 & 0.3924 & 1.0000 \end{array}\right]\times \;\left[\begin{array}{c} 0.6296\\ 0.2583\\ 0.1122 \end{array}\right]\\ \;=\;\left[\begin{array}{c} 1.8952\\ 0.7775\\ 0.3377 \end{array}\right]\\ \lambda max\;=\;\frac{1}{n}\overset{n}{\underset{i\;=\;1}{\sum }}\frac{{\left[AW\right]}_{i}}{W_{i}}\;=\;\frac{1}{3}\left(\frac{1.8952}{0.6296}+\frac{0.7775}{0.2583}+\frac{0.3377}{0.1122}\right)\\ \;=\;3.0104 \end{array}$$

⑥ Consistency test (CI) for the decision matrix.


$$\begin{array}{c} CI\;=\;\frac{\lambda \max-n}{n\;-\;1}\;=\;\frac{3.0104\;-\;3}{3\;-\;1}\;=\;0.0052\\ CR\;=\;\frac{CI}{RI}\;=\;\frac{0.0052}{0.52}\;=\;0.0100\;<\;0.1 \end{array}$$

The final calculated weights for Must-have Quality (*M*), One-dimensional Quality (O) and Attractive Quality (A) are shown in Table 9B.

**Figure 6:**
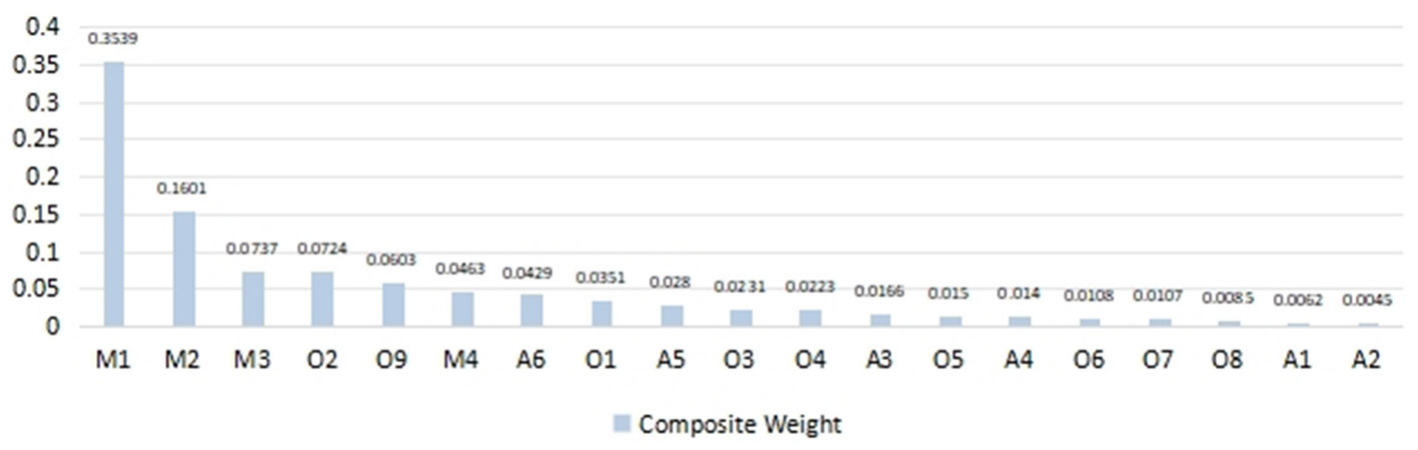
User requirement priority ranking.

Once again, the AHP (Analytic Hierarchy Process) was employed to design a questionnaire for assessing the importance of requirements at the sub-criteria level. The questionnaire continued to use a 1–9 rating scale, and a team of 15 experts was invited to score the responses, conducting pairwise comparisons of user requirements within each sub-criteria level. Based on Tables 5, 6, 7, and 8, a decision matrix was established to calculate the weight values for each type of requirement, and consistency tests were performed, as shown in Tables 10–12. The consistency ratios (*CR*) were 0.0393, 0.0058, and 0.034, respectively, all of which passed the consistency test. By calculating the composite weights of the 19 sub-requirements, we ultimately obtained the composite weights and ranking of design requirements for senior-friendly nursing home residential spaces, as shown in Table 13 and Figure 6.

**Table 10:** Must-have requirements judgment matrix and weighting.

*M*	M1	M2	M3	M4	Weight	λ_max_	*CI*	*CR*
M1	1	3.2061	4.9340	5.2733	0.5621	4.1049	0.0350	0.0393
M2	0.3119	1	2.7410	3.6553	0.2473			
M3	0.2027	0.3648	1	2.1277	0.1171			
M4	0.1896	0.2736	0.4700	1	0.0735			

**Table 11:** One-dimensional requirements judgment matrix and weights.

O	O1	O2	O3	O4	O5	O6	O7	O8	O9	Weight	λ_max_	*CI*	*CR*
O1	1	0.3852	1.8156	1.6309	2.1020	3.7350	2.8351	4.5398	0.5968	0.1359	9.0673	0.0084	0.0058
O2	2.5959	1	2.8598	3.1724	5.1560	7.7059	6.6044	7.7129	0.9810	0.2804			
O3	0.5508	0.3497	1	0.9117	2.2715	1.7215	2.0930	2.7693	0.4048	0.0895			
O4	0.6132	0.3152	1.0968	1	1.6309	1.8378	2.0873	2.4808	0.3521	0.0863			
O5	0.4757	0.1939	0.4402	0.6132	1	1.6625	1.3404	1.8378	0.3186	0.0580			
O6	0.2677	0.1298	0.5809	0.5441	0.6015	1	1.1487	1.3356	0.1682	0.0418			
O7	0.3527	0.1514	0.4778	0.4791	0.7460	0.8706	1	1.2030	0.1712	0.0416			
O8	0.2203	0.1297	0.3611	0.4031	0.5441	0.7487	0.8312	1	0.1456	0.0330			
O9	1.6756	1.0194	2.4701	2.8398	3.1390	5.9453	5.8402	6.8683	1	0.2334			

**Table 12:** Attractive requirements judgment matrix and weights.

A	A1	A2	A3	A4	A5	A6	Weight	λ_max_	*CI*	*CR*
A1	1	1.9738	0.2874	0.3879	0.1932	0.1642	1	6.1070	0.0214	0.0170
A2	0.5066	1	0.2279	0.3924	0.1639	0.1509	0.5066			
A3	3.4796	4.3876	1	1.0150	0.5503	0.3117	3.4796			
A4	2.5778	2.5487	0.9852	1	0.4418	0.3365	2.5778			
A5	5.1756	6.1016	1.8171	2.2636	1	0.4811	5.1756			
A6	6.0914	6.6278	3.2078	2.9716	2.0784	1	6.0914			

**Table 13:** Overall weighting of alternatives layer.

Criteria layer	Weight	Alternatives layer	Weight	Composite weighting	Criteria layer	Weight	Alternatives layer	Weight	Composite weighting
*M*	0.6296	M1	0.5621	0.3539	O	0.2583	O1	0.1359	0.0351
		M2	0.2473	0.1557			O2	0.2804	0.0724
		M3	0.1171	0.0737			O3	0.0895	0.0231
		M4	0.0735	0.0463			O4	0.0863	0.0223
A	0.1122	A1	0.0549	0.0062			O5	0.0580	0.0150
		A2	0.0404	0.0045			O6	0.0418	0.0108
		A3	0.1477	0.0166			O7	0.0416	0.0107
		A4	0.1247	0.0140			O8	0.0330	0.0085
		A5	0.2500	0.0280			O9	0.2334	0.0603
		A6	0.3823	0.0429					

### 3.4 PUGH design verification

Based on the results of independent interviews and questionnaire surveys conducted at the Senior Apartments in Chang'an District, Shijiazhuang, and taking into account the operational characteristics of this “urban elderly care facility” and the common needs of its residents, four distinct types of senior-friendly residential space designs have been developed. This proposal is primarily tailored for public, affordable elderly care facilities in urban areas of North China that closely resemble the study site in terms of operational models and resident demographics. Based on preliminary research and analysis, we combined the KANO model for demand classification with the AHP hierarchical analysis method for weighting demand prioritization. Centered on safety adaptation as the core, supported by functional utility, and enhanced by emotional comfort, this design principle integrates the physiological and psychological characteristics of low-, middle-, and high-age seniors (Dong and Zhu, 2026). Design four distinct residential space solutions, each focusing on different design priorities and implementation levels. These solutions are tailored to accommodate varying renovation budgets, spatial scales, and care requirements for elderly residents in nursing homes. Specific proposals are as follows:

Option 1: basic protection type.

**Figure 7:**
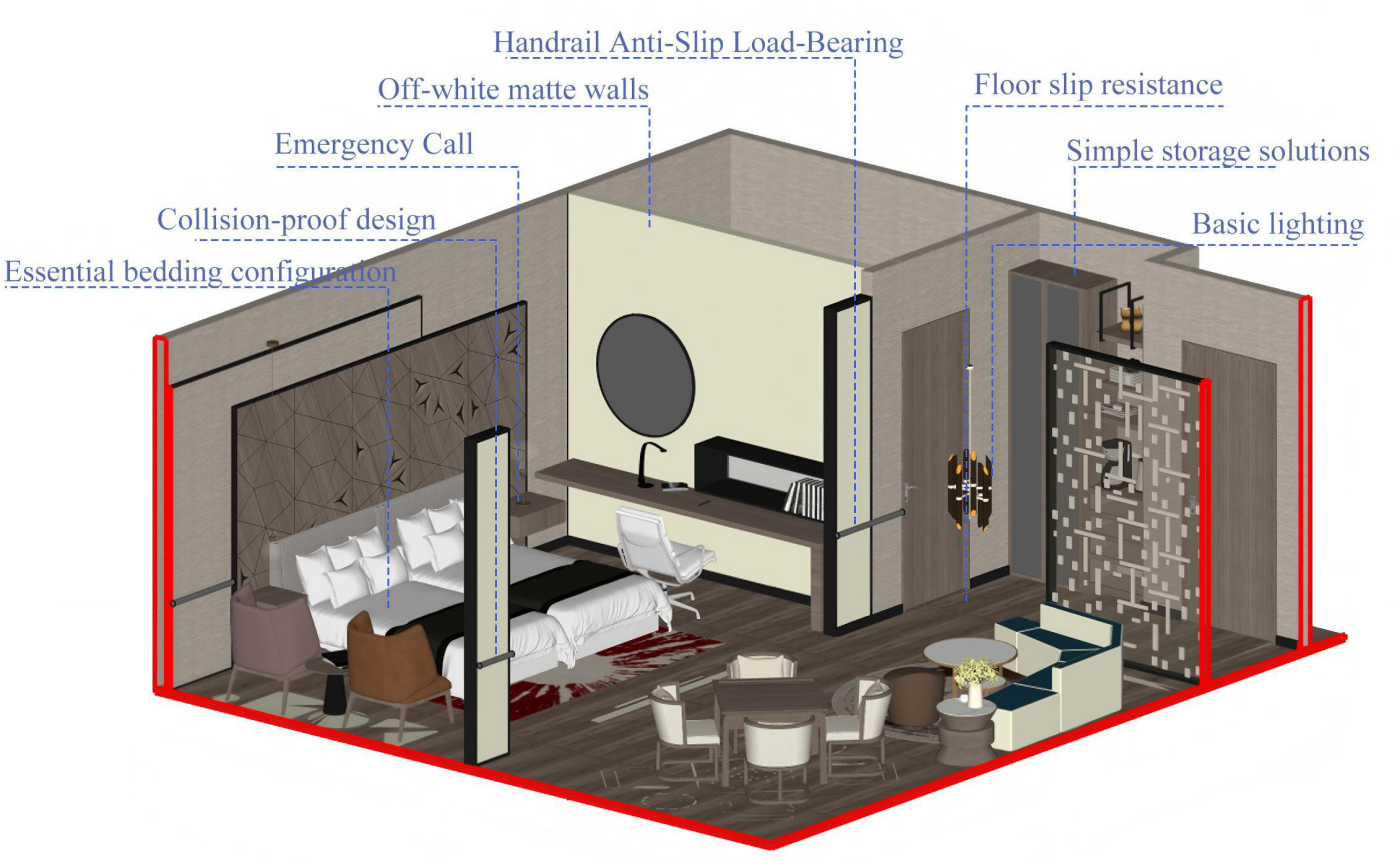
The schematic diagram of the first option.

Focusing on meeting KANO Must-have requirements (*M*) as the core. Focus on the top four core safety needs in AHP weighting, eliminate non-attractive requirements, and achieve basic aging-in-place adaptations for sleeping and residential spaces at minimal renovation cost. At the safety adaptation level, implement core requirements such as emergency call functionality, rounded corner collision prevention design, and anti-slip load-bearing handrails with basic floor slip resistance. At the functional utility level, simplify the implementation of basic lighting, simple storage solutions, and essential bedding configuration. The Emotional Comfort Level provides only the basic environment of off-white matte walls, with no additional greenery, nostalgic elements, or privacy partitions. This option features the lowest overall renovation cost, minimal construction complexity, and a short implementation timeline. Its core focus is on “safety and accident prevention,” making it suitable for budget-conscious, inclusive nursing homes and areas housing younger seniors in relatively good health. The schematic diagram of the first option is shown in Figure 7.

Option 2: standard adaptation type.

**Figure 8:**
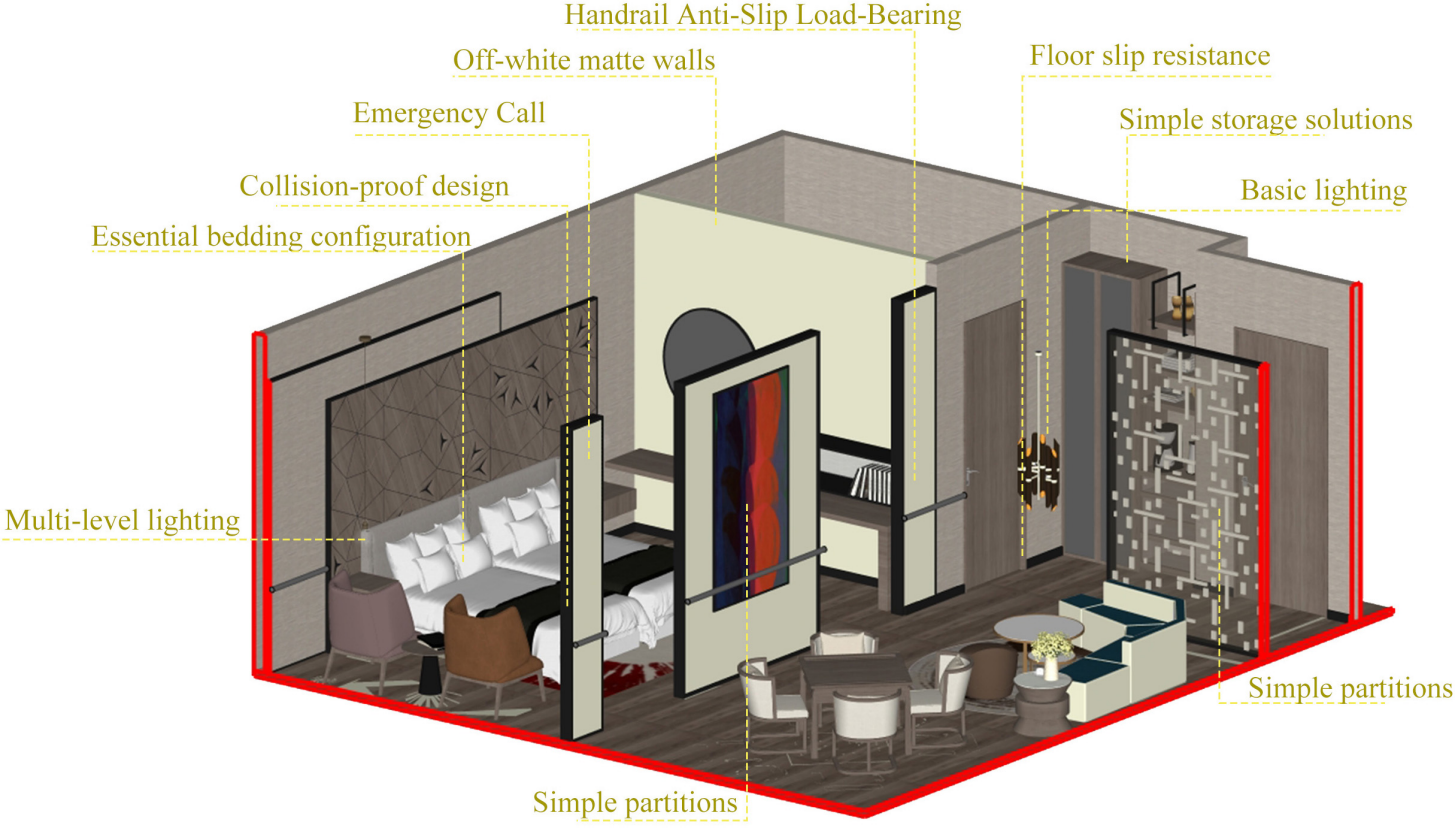
The schematic diagram of the secondly option.

The Standard Adaptive Solution targets mid-range nursing homes, providing comprehensive care coverage at a moderate cost. The design centers on essential and high-priority expectations derived from the KANO model, implementing the top 10 indicators weighted by AHP while balancing safety, practicality, and fundamental emotional comfort. For safety, it features one-touch emergency call, rounded corners for collision prevention, motion-activated night lights, and electrical shock protection covers. Additionally, it achieves seamless, level pathways throughout the home and incorporates slip-resistant, durable flooring. The design centers on essential and high-priority expectations derived from the KANO model, implementing the top 10 indicators weighted by AHP while balancing safety, practicality, and fundamental emotional comfort. For safety, it features one-touch emergency call, rounded corners for collision prevention, motion-activated night lights, and electrical shock protection covers. Additionally, it achieves seamless, level pathways throughout the home and incorporates slip-resistant, durable flooring. Functionally, it features multi-level lighting, close-range storage, adjustable bedding, and basic temperature control as standard. Emotionally, it employs warm off-white and light beige tones complemented by greenery and simple partitions to soften the institutional feel. This design eliminates redundancy, offers moderate renovation costs, and aligns with the practical living needs of elderly residents. The schematic diagram of the secondly option is shown in Figure 8.

Option 3: quality enhancement type.

**Figure 9:**
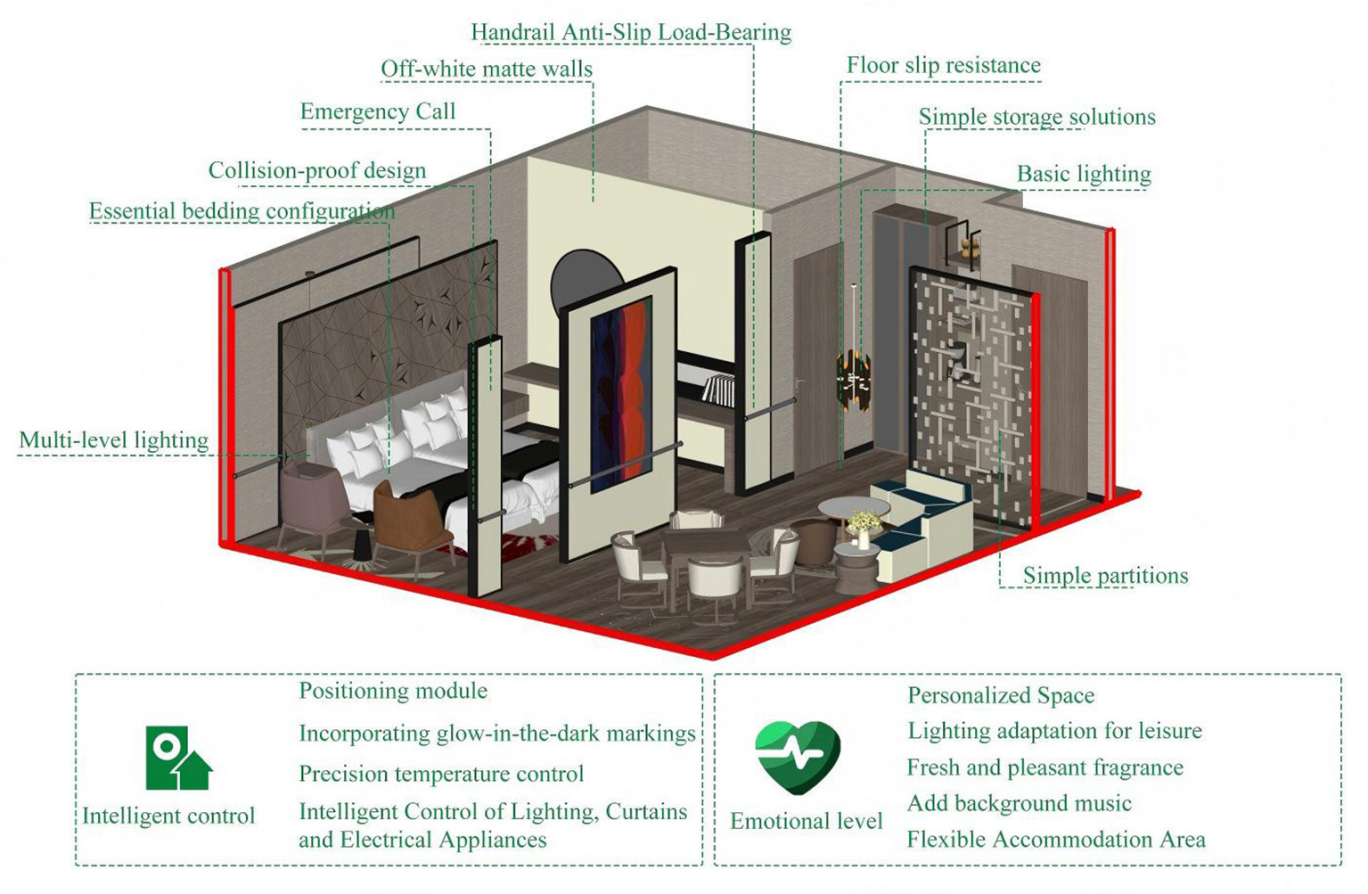
The schematic diagram of the thirdly option.

The Quality Enhancement Solution is specifically designed for high-end nursing homes, accommodating higher renovation costs and refined care requirements. The solution centers on the KANO model's must-have requirements, fully expected requirements, and high-weight appeal requirements, comprehensively covering the top 15 indicators by AHP weight. Building upon safety and functionality, we enhance emotional comfort and personalized experiences. On the safety front, we upgrade essential features such as adding a positioning module to the emergency call system and incorporating glow-in-the-dark markings on the armrests. Functionally, it enables smart control of lighting, curtains, and appliances while implementing precise temperature and humidity regulation. Emotionally, it fulfills high-priority needs such as personalized spaces, leisure-adapted lighting, fresh and comfortable scents, and flexible companion zones, complemented by background music to cultivate a tranquil atmosphere. Though costly, this meticulously designed solution balances care accessibility with seniors' psychological needs, precisely aligning with the quality-driven development of premium senior living facilities. The schematic diagram of the thirdly option is shown in Figure 9.

Option 4: smart integration type.

**Figure 10:**
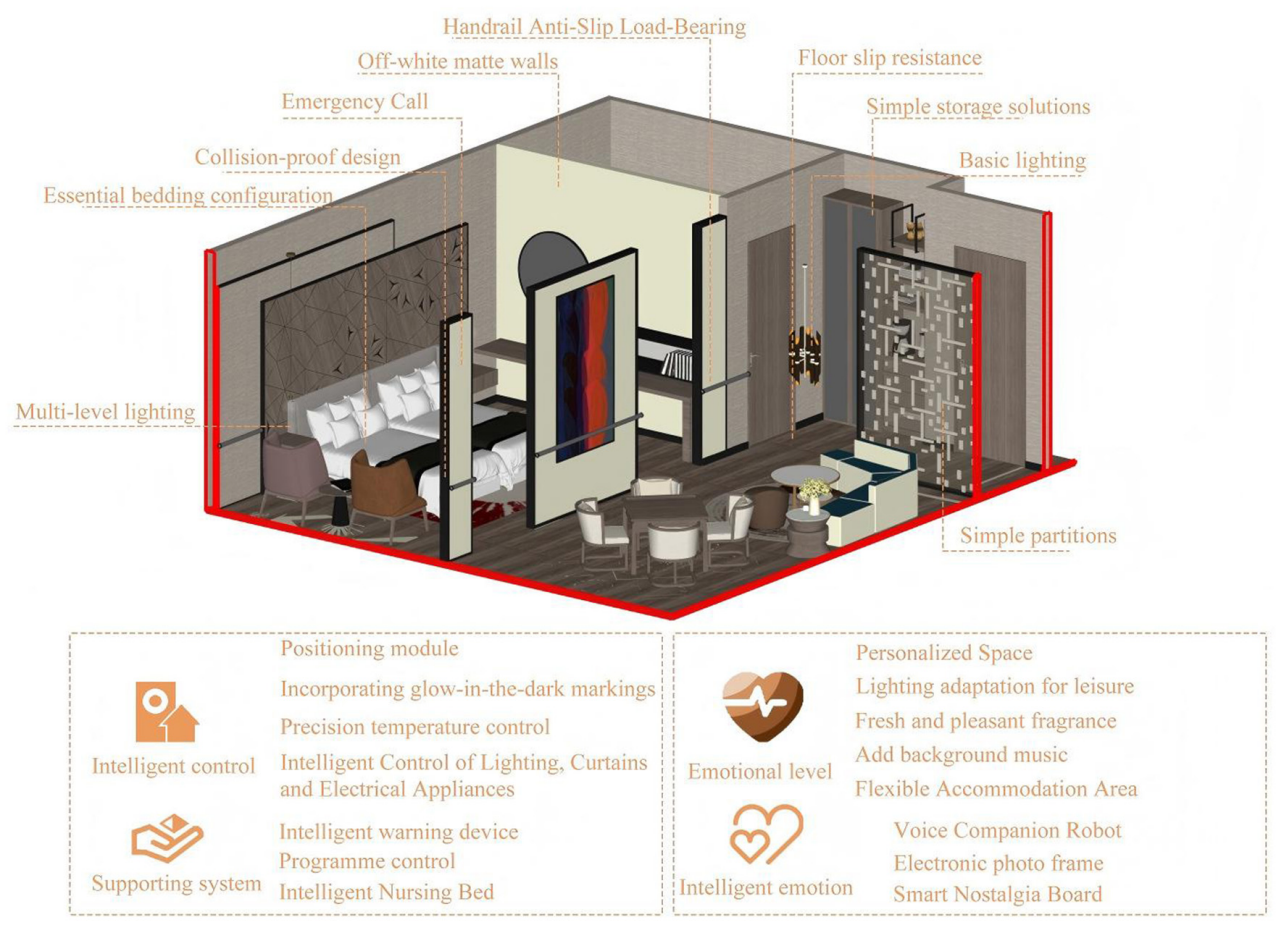
The schematic diagram of the fourthly option.

The Smart Integration Solution targets premium smart senior care facilities, addressing high renovation costs and intelligent care requirements. Building upon the Quality Enhancement Solution, it incorporates smart aging-in-place technologies centered on “Safety + Functionality + Emotional Support + Intelligence” to achieve a smart upgrade of residential spaces. On the safety front, the original design has been enhanced with intelligent warning devices such as pressure-sensitive anti-slip mats and human presence sensors. Functionally, it enables voice and remote control of lighting, curtains, and appliances, featuring smart nursing beds with lift-assist and pressure-sore prevention capabilities. Emotionally, it incorporates digital photo frames, smart memory boards, and voice companion robots to enhance personalized experiences and companionship through intelligent means. This solution represents the highest renovation cost with outstanding technological sophistication, driving a shift from passive to proactive care models aligned with smart nursing home development trends (Nedeljko et al., 2023). The schematic diagram of the fourthly option is shown in Figure 10.

#### 3.4.1 Preliminary screening of design proposals

The PUGH decision matrix is a rapid decision-making method that aids in identifying optimal design solutions (Guler and Petrisor, 2021). During the design formulation process, we first employ the PUGH decision matrix for preliminary screening. Subsequently, shortlisted proposals undergo comprehensive evaluation to determine the optimal candidate. Based on the evaluation results, we implement iterative optimization until the final design solution is established.

We selected 3 nursing home staff members, 5 environmental design professionals, and 4 graduate students specializing in age-friendly design to form a 12-member proposal review panel. All members of the review panel underwent standardized training in advance on the KANO model for requirement classification, AHP weighting analysis, and the Pugh decision matrix review method used in this study, to ensure consistent evaluation criteria and objective results. The review panel conducted preliminary evaluations of four design proposals for senior-friendly residential spaces in nursing homes. We will use Option 2: standard adaptation type as the baseline solution and compare it against the other available options. Record “+1” for solutions outperforming the baseline, “0” for solutions matching the baseline, and “−1” for solutions underperforming the baseline. Tally the solution scores, calculate the net scores for each solution, and rank them accordingly. The comprehensive evaluation and results are presented in Table 14.

**Table 14:** Preliminary scoring and ranking of design schemes for elderly-friendly nursing home residential spaces.

Evaluation criteria	Option one	Option two	Option three	Option four
Must-have quality (*M*)	0	0	0	0
One-dimensional quality (O)	−1	0	+1	+1
Attractive quality (A)	−1	0	+1	+1
Total score	−2	0	+2	+2
Sort order	3	2	1	1
Result	Abandon	Retain	Retain	Retain

#### 3.4.2 Comprehensive evaluation of design options

Following the initial screening of proposals, we have retained Proposals Two, Three, and Four for further comprehensive evaluation. The comprehensive evaluation continues to use Option 2 as the baseline. We assessed each user requirement on a five-point scale ranging from 1 to 5. Option 2 received a score of 3 across all metrics. After collecting scores for Options 3 and 4, we calculated the corresponding demand value scores for each metric by applying the respective comprehensive weighting values. The results are shown in Table 15.

**Table 15:** Value scores for design requirements of elderly-friendly nursing home residential spaces.

Indicator	Weight	Option two		Option three		Option four	
		Score	Comprehensive score	Score	Comprehensive score	Score	Comprehensive score
M1	0.3539	3	1.0737	4	1.4156	4	1.4156
M2	0.1557	3	0.4803	4	0.6228	4	0.6228
M3	0.0737	3	0.2283	4	0.2948	5	0.3685
M4	0.0463	3	0.1890	4	0.1852	4	0.1852
O1	0.0351	3	0.2232	4	0.1404	4	0.1404
O2	0.0724	3	0.1254	5	0.3620	5	0.3620
O3	0.0231	3	0.1086	4	0.0924	4	0.0924
O4	0.0223	3	0.1098	4	0.0892	4	0.0892
O5	0.0150	3	0.0822	5	0.0750	5	0.0750
O6	0.0108	3	0.0714	4	0.0432	4	0.0432
O7	0.0107	3	0.0678	4	0.0428	5	0.0535
O8	0.0085	3	0.0489	5	0.0425	5	0.0425
O9	0.0603	3	0.0441	4	0.2412	5	0.3015
A1	0.0062	3	0.0411	5	0.0310	5	0.0310
A2	0.0045	3	0.0288	4	0.0180	4	0.0180
A3	0.0166	3	0.0285	4	0.0664	5	0.0830
A4	0.0140	3	0.0198	4	0.0560	4	0.0560
A5	0.0280	3	0.0174	4	0.1120	4	0.1120
A6	0.0429	3	0.0120	4	0.1716	5	0.2145
Total Score	3.0000	4.1021	4.4063				

Based on the comprehensive scoring results in Table 15, we evaluate Solution 3 and Solution 4 from the perspective of implementation feasibility. The implementation feasibility dimension is broken down into four metrics: controllability of renovation costs, construction adaptability, operational and maintenance convenience, and care adaptability and universality. These metrics primarily assess the practicality of the solution throughout the entire process from design to implementation. The results are shown in Table 16. We continue to evaluate the four project indicators using a five-point scale ranging from 1 to 5. Each indicator carries a 10% weighting, while the combined weighting for the demand value score and implementation feasibility score is 60 and 40%, respectively.

**Table 16:** Scoring of design schemes for elderly-friendly nursing home residential spaces.

Indicator dimensions		Composite weighting		Option two	Option three	Option four
Demand value score	0.60	3.0000	4.1021	4.4063		
Feasibility score	Controllability of renovation costs	0.40	0.10	3	3	2
	Construction adaptability		0.10	3	4	3
	Operational and maintenance convenience		0.10	3	4	2
	Care adaptability and universality		0.10	3	4	2
Overall score	1.00	3.000	3.9613	3.5438		

As shown in Table 16, which evaluates the design proposals for senior-friendly residential spaces in nursing homes, Proposal 3 achieved a higher overall feasibility score than Proposal 4. Although Proposal 4 incorporates innovative smart features, its implementation is constrained by cost, compatibility, and operational maintenance requirements. Consequently, it is only suitable for high-end smart nursing homes with ample funding, technological resources, and specialized maintenance teams. As the optimal solution for a specific niche scenario, it lacks universal applicability for widespread implementation.

## 4 Discussion

### 4.1 Presentation of key findings

This study systematically completed the demand exploration, classification, prioritization, and solution screening for elderly-friendly residential spaces in nursing homes through an integrated multi-model approach combining “Empathy Map-KANO-AHP-PUGH”. The core research findings are as follows: first, through semi-structured interviews and empathy mapping, we precisely identified 22 core needs for the sleeping and residential spaces of elderly residents in nursing homes. These needs span three dimensions: safety and accessibility, functional utility, and emotional comfort. This approach addresses the shortcomings of traditional research, which often fails to adequately uncover implicit emotional needs.

Second, based on the KANO model, requirements are categorized into three types: must-have Requirements (*M*), One-dimensional Requirements (O) and Attractive Requirements (A). Among these, six features—Emergency Call (Q4) and Collision-proof design (Q5)—are Must-have Requirements. Nine features—No elevation difference in the flow path (Q1) and Handrail Anti-Slip Load-Bearings (Q2)—are One-dimensional Requirements. Seven features—Power Supply Electric Shock Protection (Q7) and Personalized Space (Q15)—are Attractive Requirements. Third, the AHP hierarchical analysis method determined the weighting order of criteria levels as follows: must-have Requirements (*M*) > One-dimensional Requirements (O) > Attractive Requirements (A). At the alternatives layer, emergency call (M1), rounded anti-collision design (M2), and non-slip load-bearing handrails (O2) are core priority requirements. Fourth, through preliminary screening via the PUGH strategy matrix and comprehensive quantitative evaluation, the quality enhancement approach emerged as the core optimal solution with the highest overall score. The intelligent integration approach, constrained by implementation limitations, was identified only as the optimal solution for specific scenarios.

Regarding demand prioritization, the high-weight essential needs identified in this study corroborate the assessment logic of “fundamental safety assurance as the core” outlined in the “Classification and Evaluation of Elderly Care Institutions”. The weighting allocation between one-dimensional and attractive needs aligns with the core tenets of the KANO model—where explicit needs linearly enhance satisfaction while implicit needs create surprising experiences—while also enabling a scientific prioritization of needs through AHP quantification. This approach avoids the subjective biases inherent in designer-led experience-driven methodologies (Liu et al., 2020). This aligns with the integrated “KANO-AHP-QFD” approach adopted by Fang et al. in designing medication reminder applications for the elderly (Fang et al., 2023), but further refines the demand weighting system for spatial dimensions within the field of age-friendly spaces.

The screening results indicate that the quality enhancement approach excels in achieving “comprehensive coverage of requirements balanced with implementation feasibility.” This finding aligns with existing research advocating that “age-friendly design must balance scientific rigor with practicality” (Xue et al., 2025). Although the smart integration approach had a slightly higher perceived value, its overall rating was lower due to practical constraints such as renovation costs and technical barriers. While the smart integration solution scored slightly higher in terms of perceived value, its application scenarios were clearly limited when implementation constraints, user acceptance and resource considerations were taken into account. In terms of implementation constraints, this solution involves integrating smart devices with existing smart systems in senior care facilities through hardware and software interfaces. The renovation process faces technical challenges such as ensuring device compatibility and modifying spatial wiring and piping. In terms of user acceptance, older adults generally have little inclination to learn how to use new devices, which makes it difficult to utilize smart devices flexibly. From a resource perspective, the per-room renovation cost of the smart integration solution is approximately 2.5 to 3 times higher than that of the quality enhancement solution. Furthermore, daily maintenance and future upgrades of smart devices require a professional technical team and sustained financial investment, presenting dual constraints of funding and operational resources for affordable, grassroots elderly care facilities. This suggests that age-friendly design should avoid “technological worship” (Ghorayeb et al., 2021) and instead focus on real-world usage scenarios for seniors and the operational realities of nursing homes (Zhao and Li, 2024).

### 4.2 Discussion of findings and theoretical dialogue

The multi-model integration approach employed in this study establishes a decision-making process for age-friendly spatial design, spanning from needs analysis to the implementation of design solutions, thereby fostering meaningful dialogue with existing research and driving innovative advancements. Existing studies predominantly employ either the KANO or AHP model for needs analysis, lacking in-depth exploration of latent needs and multidimensional solution validation. The“Empathy Map-KANO-AHP-PUGH” integrated model developed in this study represents a significant breakthrough and extension of existing research in the field of senior-friendly space design. First, regarding the depth of needs assessment, existing studies typically rely on single questionnaires or interviews to identify the needs of older adults, focusing solely on explicit needs such as physical safety. In contrast, this study incorporates the Empathy Map into the preliminary needs assessment phase. By adopting a four-dimensional perspective—“Feelings – Behaviors – Thoughts - Visual Experience,“ this study transforms implicit psychological needs—such as feelings of loneliness and privacy requirements—into quantifiable design metrics, thereby addressing the shortfall in existing research regarding the exploration of implicit needs (Higuera and Macías, 2024). This is fully consistent with the research findings that “age-friendly environments must address both physical and psychological needs”(Au et al., 2020). Second, existing research on multi-model integration has primarily focused on areas such as assistive devices for the elderly and smart aging-in-place products, with few empirical studies applied to the residential spaces within senior care facilities. By integrating qualitative empathy with quantitative decision-making, this approach provides more objective and systematic decision-making guidance for the design of age-friendly residential spaces in senior care facilities.

Although this study has established a relatively comprehensive decision-making process, it must be noted that the final design has not been empirically validated; no post-occupancy evaluations, real-world user testing, long-term behavioral observations, or iterative optimization have been conducted. Therefore, the contribution of this study should be viewed as a preliminary integrated decision-support framework, rather than a mature design system that has been fully tested in practice and is ready for direct replication and implementation.

### 4.3 Practical significance

The results of the screening for quality-enhancement solutions provide direct practical guidance for age-friendly renovations of similar senior care facilities in North China. Their modular design adapts to various types of nursing homes—from affordable to mid-range and high-end—providing practical implementation models for policy execution. The solutions emphasize safety upgrades, functional optimization, and emotional empowerment. These elements not only align with the requirements of the “14th Five-Year Plan for the Development of the Elderly Care Service System” but also provide an environmental optimization pathway to alleviate rest barriers for the elderly and enhance the quality of home-based care.

Our research findings indicate that the integrated Empathy Map-KANO-AHP-PUGH model can be effectively applied to age-friendly space design, thereby enhancing user satisfaction. Compared to traditional design approaches reliant on designer experience, this method offers greater precision in capturing users' unique needs. Compared to previous research, the Empathy Map-KANO-AHP-PUGH methodology offers two key advantages: first, the Empathy Map effectively uncovers the latent needs of the elderly population. Combined with the Kano model, it enables design teams to efficiently identify user requirements, ensuring design elements precisely align with user expectations and demands. Secondly, the Analytic Hierarchy Process (AHP) enables detailed analysis of each user requirement. By deeply integrating design elements with user needs, it significantly optimizes the user experience. Furthermore, the Pugh Matrix provides a powerful tool for implementing optimized design solutions, capable of selecting the best option from multiple design proposals.

## 5 Conclusion

### 5.1 Research summary

This study aims to alleviate rest-related challenges among the elderly and enhance the quality of life in home-based care. The Chang'an Community Senior Apartments in Shijiazhuang were selected as the research site. Through semi-structured interviews with 40 seniors and stratified sampling using the MMSE, multiple methodologies—including empathy map, the KANO model, the AHP hierarchical analysis method, and the PUGH decision matrix—were integrated. This approach enabled the identification of needs, classification and prioritization, design proposals, and quantitative evaluation for senior-friendly residential spaces within nursing homes. This study identified 22 core needs for senior residential spaces. Based on the KANO model, three irrelevant and reverse-type needs with no design value were eliminated, resulting in the final selection of 19 valid indicators, which were then weighted and ranked. This process led to the development of four distinct design schemes. Developing four differentiated design solutions: foundational support, standard adaptation, quality enhancement, and intelligent integration. Ultimately, the quality enhancement solution—which achieves “full coverage of core requirements with high implementation feasibility”—was selected as the optimal core solution. Due to technical implementation constraints, varying levels of acceptance among elderly users, and limited resource allocation in senior care facilities, this intelligent integration solution is currently the optimal solution for specific scenarios—namely, high-end smart senior care facilities that possess a comprehensive smart infrastructure, ample funding and operational resources, and primarily serve younger seniors with high cognitive abilities. However, due to these multiple factors, the conditions for widespread adoption in ordinary senior care facilities are not yet in place.

### 5.2 Strengths and limitations

The strengths of this study are primarily reflected in three aspects: first, methodological innovation—it established an integrated full-process system encompassing “hidden needs exploration, quantitative needs classification, and multidimensional solution evaluation,” enhancing the scientific rigor and precision of age-friendly design. Second, the representative sample ensures the universality of findings through MMSE-based cognitive stratification and gender-balanced sampling. Third, the strong practical applicability of outcomes integrates design solutions with the architectural characteristics and operational realities of aging nursing homes, bridging the gap between theory and practice.

This study employed an integrated approach to develop a decision-making framework for nursing home living spaces and formulate an optimal design scheme; however, it still has the following limitations, which future research should address. First, the sample scope was limited to Chang'an District in Shijiazhuang, and did not include nursing homes in different regions or areas with varying economic levels. As demand characteristics may vary by region, the level of detail in the demand analysis needs to be improved (Oswald et al., 2007). Second, both the AHP (Analytic Hierarchy Process) and the PUGH (Program Evaluation and Ranking of Alternatives) methods used in this study rely on expert scoring. Although objectivity is enhanced through multi-expert aggregation and consistency checks, the experts' professional backgrounds, experience, preferences, and value judgments may still introduce some degree of subjective bias. Third, this study has only completed theoretical-level needs analysis and scheme comparison; the final design scheme has not undergone post-occupancy evaluation (POE), user testing, behavioral observation, or verification of actual usage effectiveness. In the future, we will conduct post-occupancy evaluations, user testing, and long-term behavioral tracking studies to verify the practical effectiveness of the design scheme and further refine this decision-support framework (Petri et al., 2025). Fourth, the conclusions of this study are based on community-based elderly care facilities in northern cities, where spatial conditions, operational models, and the structure of elderly residents' needs exhibit significant contextual specificity. Therefore, these findings cannot be directly generalized to different settings, such as rural elderly care facilities, and their applicability is clearly limited.

### 5.3 Future research prospects

Future research can be expanded in three directions: first, expand the sample scope by conducting large-scale, independent surveys that span different regions and business models, thereby establishing a more universally applicable system for weighting consumer needs. Second, deepen the analysis of individual differences by conducting segmented demand studies for seniors of different genders, disability levels, and living habits, designing more personalized adaptation solutions. Third, conduct long-term effectiveness tracking by implementing quality-enhancement pilot programs in real settings. Collect dynamic data on resident satisfaction and care efficiency to validate the sustained efficacy of solutions and identify optimization opportunities. Additionally, integrate IoT and big data technologies to explore dynamic monitoring of aging-friendly spatial needs and intelligent iteration mechanisms for design solutions, advancing aging-in-place design toward precision and intelligence.

## Data Availability

The original contributions presented in the study are included in the article/Supplementary material, further inquiries can be directed to the corresponding author.
