# Supplementary material for: Specific design strategies for elderly-friendly residential spaces in nursing homes: an integrated model approach based on empathy map-KANO-AHP-PUGH
Source: Front Psychol. 2026 May 11;17:1824564. doi: 10.3389/fpsyg.2026.1824564 (PMC13199012; doi:10.3389/fpsyg.2026.1824564)
Supplement: Supplementary file 1 [file Data_Sheet_1.pdf]

Appendix：

Specific Interview Questions Table

Dimension	Interview keywords	Summary
Spatial Layout Adaptability		
	"Narrow passageways", "prone to collisions", "excessively sharp corridor corners", "mutual interference"	Inadequate circulation design
	"Clothing is haphazardly piled", "lack of privacy", "no simple activity area"	Function area is blurred
	Lack of nearby storage space for items, and cluttering of items affecting lighting and ventilation	Low space utilization
Functional facility compatibility		
	"Stimulating both eyes with nighttime lighting", "Difficulty in turning on lights at night", "Inconvenience of getting up at night"	Design Defects of Lighting Facilities
	"no support point", "slippery"	Lack or incompatibility of auxiliary facilities
	"Direct air conditioning blow" and "Temperature fluctuation"	Insufficient temperature control and ventilation design
Environmental compatibility		
	Poor sound insulation, noise at night	Insufficient sound insulation and privacy
	"The ground is too bright" and "Lacks a sense of warmth"	Visual and tactile discomfort
	"Single decoration style", "lack of green plants", "loneliness"	Lack of emotional adaptation design

Questionnaire on the Importance of Indicators in the Analytic Hierarchy Process

Expert 1:

Importance Matrix for First-Level Indicators:

Requirement Type	Must-have Quality(M)	One-dimensional Quality(O)	Attractive Quality(A)
Must-have Quality(M)	1	5	4
One-dimensional	1/5	1	1/2

Quality(O)			
Attractive Quality(A)	1/4	2	1

Matrix for Assessing the Importance of Secondary Indicators:

Must-have Quality(M)

Must-have Quality(M)	M1	M2	M3	M4
M1	1	5	5	9
M2	1/5	1	4	5
M3	1/5	1/4	1	2
M4	1/9	1/5	1/2	1

One-dimensional Quality(O)

One-dimensional Quality(O)	O1	O2	O3	O4	O5	O6	O7	O8	O9
O1	1	1/2	1/2	2	3	5	3	2	1/2
O2	2	1	4	4	6	8	8	9	1
O3	2	1/4	1	1	2	1	3	5	1/2
O4	1/2	1/4	1	1	2	1/4	2	5	1/3
O5	1/3	1/6	1/2	1/2	1	1/2	1/2	3	1/4
O6	1/5	1/8	1	4	2	1	2	2	1/7
O7	1/3	1/8	1/3	1/2	2	1/2	1	2	1/4
O8	1/2	1/9	1/5	1/5	1/3	1/2	1/2	1	1/9
O9	2	1	2	3	4	7	4	9	1

Attractive Quality(A)

Attractive Quality(A)	A1	A2	A3	A4	A5	A6
A1	1	2	1/3	1/3	1/5	1/7
A2	1/2	1	1/6	1/2	1/7	1/9
A3	3	6	1	1	1/2	1
A4	3	2	1	1	1/3	1/4
A5	5	7	2	3	1	1/5
A6	7	9	1	4	5	1

Expert 2:

Importance Matrix for First-Level Indicators:

Requirement Type	Must-have Quality(M)	One-dimensional Quality(O)	Attractive Quality(A)
Must-have Quality(M)	1	1	5
One-dimensional Quality(O)	1	1	8
Attractive Quality(A)	1/5	1/8	1

Matrix for Assessing the Importance of Secondary Indicators:

Must-have Quality(M)

Must-have Quality(M)	M1	M2	M3	M4
M1	1	6	9	9
M2	1/6	1	1	5
M3	1/9	1	1	4
M4	1/9	1/5	1/4	1

One-dimensional Quality(O)

One-dimensional Quality(O)	O1	O2	O3	O4	O5	O6	O7	O8	O9
O1	1	1/4	3	2	5	4	4	3	1/2
O2	4	1	5	4	8	9	5	9	2
O3	1/3	1/5	1	1/4	3	1/2	2	1/5	1/4
O4	1/2	1/4	4	1	2	2	3	3	1/4
O5	1/5	1/8	1/3	1/2	1	2	3	1/2	1/4
O6	1/4	1/9	2	1/2	1/2	1	1	1	1/7
O7	1/4	1/5	1/2	1/3	1/3	1	1	1/2	1/9
O8	1/3	1/9	5	1/3	2	1	2	1	1/9
O9	2	1/2	4	4	4	7	9	9	1

Attractive Quality(A)

Attractive Quality(A)	A1	A2	A3	A4	A5	A6
A1	1	2	1/3	1/2	1/7	1/8
A2	1/2	1	1/6	1/3	1/9	1/9
A3	3	6	1	1	1	1/6
A4	2	3	1	1	1/2	1
A5	7	9	1	2	1	1/3
A6	8	9	6	1	3	1

Expert 3:

Importance Matrix for First-Level Indicators:

Requirement Type	Must-have Quality(M)	One-dimensional Quality(O)	Attractive Quality(A)
Must-have Quality(M)	1	4	9
One-dimensional Quality(O)	1/4	1	1
Attractive Quality(A)	1/9	1	1

Matrix for Assessing the Importance of Secondary Indicators:

Must-have Quality(M)

Must-have Quality(M)	M1	M2	M3	M4
M1	1	1	2	6
M2	1	1	3	7
M3	1/2	1/3	1	9
M4	1/6	1/7	1/9	1

One-dimensional Quality(O)

One-dimensional Quality(O)	O1	O2	O3	O4	O5	O6	O7	O8	O9
O1	1	1/2	5	2	2	5	5	4	1
O2	2	1	3	4	5	8	8	9	1/2
O3	1/5	1/3	1	1	2	1	2	5	2
O4	1/2	1/4	1	1	2	2	2	4	1/3
O5	1/2	1/5	1/2	1/2	1	2	3	3	1/2
O6	1/5	1/8	1	1/2	1/2	1	2	2	1/4
O7	1/5	1/8	1/2	1/2	1/3	1/2	1	2	1/7
O8	1/4	1/9	1/5	1/4	1/3	1/2	1/2	1	1/4
O9	1	2	1/2	3	2	4	7	4	1

Attractive Quality(A)

Attractive Quality(A)	A1	A2	A3	A4	A5	A6
A1	1	2	1/2	1/4	1/6	1/3
A2	1/2	1	1/6	1/2	1/7	1/9
A3	2	6	1	2	1/2	1/7

A4	4	2	1/2	1	1/2	1/5
A5	6	7	2	2	1	1/3
A6	3	9	7	5	3	1

Expert 4:

Importance Matrix for First-Level Indicators:

Requirement Type	Must-have Quality(M)	One-dimensional Quality(O)	Attractive Quality(A)
Must-have Quality(M)	1	3	2
One-dimensional Quality(O)	1/3	1	1
Attractive Quality(A)	1/2	1	1

Matrix for Assessing the Importance of Secondary Indicators:

Must-have Quality(M)

Must-have Quality(M)	M1	M2	M3	M4
M1	1	8	7	9
M2	1/8	1	2	5
M3	1/7	1/2	1	2
M4	1/9	1/5	1/2	1

One-dimensional Quality(O)

One-dimensional Quality(O)	O1	O2	O3	O4	O5	O6	O7	O8	O9
O1	1	1/2	2	2	2	3	1/2	7	1/3
O2	2	1	2	3	4	9	4	9	1
O3	1/2	1/2	1	2	2	3	2	2	1/3
O4	1/2	1/3	1/2	1	2	2	3	4	1/5
O5	1/2	1/4	1/2	1/2	1	2	3	2	1/4
O6	1/3	1/9	1/3	1/2	1/2	1	1	2	1/3
O7	2	1/4	1/2	1/3	1/3	1	1	6	1/8
O8	1/7	1/9	1/2	1/4	1/2	1/2	1/6	1	1/4
O9	3	1	3	5	4	3	8	4	1

Attractive Quality(A)

Attractive Quality(A)	A1	A2	A3	A4	A5	A6
A1	1	1	1/4	1/3	1/6	1/6
A2	1	1	1/4	1/2	1/6	1/2
A3	4	4	1	1/2	1/2	1/5
A4	3	2	2	1	1/3	1/4
A5	6	6	2	3	1	1/2
A6	6	2	5	4	2	1

Expert 5:

Importance Matrix for First-Level Indicators:

Requirement Type	Must-have Quality(M)	One-dimensional Quality(O)	Attractive Quality(A)
Must-have Quality(M)	1	2	8
One-dimensional Quality(O)	1/2	1	6
Attractive Quality(A)	1/8	1/6	1

Matrix for Assessing the Importance of Secondary Indicators:

Must-have Quality(M)

Must-have Quality(M)	M1	M2	M3	M4
M1	1	3	6	9
M2	1/3	1	5	9
M3	1/6	1/5	1	2
M4	1/9	1/9	1/2	1

One-dimensional Quality(O)

One-dimensional Quality(O)	O1	O2	O3	O4	O5	O6	O7	O8	O9
O1	1	1/2	2	2	4	4	5	6	4
O2	2	1	3	4	5	8	9	9	2
O3	1/2	1/3	1	1	2	1/3	2	4	1/4
O4	1/2	1/4	1	1	2	2	3	4	1/3
O5	1/4	1/5	1/2	1/2	1	2	1/2	2	1/3
O6	1/4	1/8	3	1/2	1/2	1	2	2	1/8
O7	1/5	1/9	1/2	1/3	2	1/2	1	2	1/8

O8	1/6	1/9	1/4	1/4	1/2	1/2	1/2	1	1/9
O9	1/4	1/2	4	3	3	8	8	9	1

Attractive Quality(A)

Attractive Quality(A)	A1	A2	A3	A4	A5	A6
A1	1	1	1/4	1/4	1/5	1/8
A2	1	1	1/3	1/2	1/8	1/9
A3	4	3	1	1/2	1/3	1/6
A4	4	2	2	1	1/2	1/6
A5	5	8	3	2	1	1/2
A6	8	9	6	6	2	1

Expert 6:

Importance Matrix for First-Level Indicators:

Requirement Type	Must-have Quality(M)	One-dimensional Quality(O)	Attractive Quality(A)
Must-have Quality(M)	1	6	9
One-dimensional Quality(O)	1/6	1	2
Attractive Quality(A)	1/9	1/2	1

Matrix for Assessing the Importance of Secondary Indicators:

Must-have Quality(M)

Must-have Quality(M)	M1	M2	M3	M4
M1	1	5	9	9
M2	1/5	1	3	7
M3	1/9	1/3	1	3
M4	1/9	1/7	1/3	1

One-dimensional Quality(O)

One-dimensional Quality(O)	O1	O2	O3	O4	O5	O6	O7	O8	O9
O1	1	1/2	1	2	2	4	5	7	1/4
O2	2	1	3	5	3	9	9	9	1/3
O3	1	1/3	1	1	2	3	5	4	2

O4	1/2	1/5	1	1	3	2	2	3	1
O5	1/2	1/3	1/2	1/3	1	2	2	2	1/2
O6	1/4	1/9	1/3	1/2	1/2	1	1	1	1/9
O7	1/5	1/9	1/5	1/2	1/2	1	1	1/2	1/5
O8	1/7	1/9	1/4	1/3	1/2	1	2	1	1/2
O9	4	3	1/2	1	2	9	5	2	1

Attractive Quality(A)

Attractive Quality(A)	A1	A2	A3	A4	A5	A6
A1	1	7	1/4	1/2	1/7	1/8
A2	1/7	1	1/3	1/4	1/5	1/9
A3	4	3	1	2	1/2	1/3
A4	2	4	1/2	1	1/3	1/4
A5	7	5	2	3	1	1
A6	8	9	3	4	1	1

Expert 7:

Importance Matrix for First-Level Indicators:

Requirement Type	Must-have Quality(M)	One-dimensional Quality(O)	Attractive Quality(A)
Must-have Quality(M)	1	1/2	1/2
One-dimensional Quality(O)	2	1	1
Attractive Quality(A)	2	1	1

Matrix for Assessing the Importance of Secondary Indicators:

Must-have Quality(M)

Must-have Quality(M)	M1	M2	M3	M4
M1	1	1/2	7	5
M2	2	1	7	9
M3	1/7	1/7	1	3
M4	1/5	1/9	1/3	1

One-dimensional Quality(O)

One-dimensional	O1	O2	O3	O4	O5	O6	O7	O8	O9

Quality(O)									
O1	1	1/5	1	1	2	4	1/3	6	1
O2	5	1	1	4	4	9	9	9	3
O3	1	1	1	1	2	4	2	5	1/2
O4	1	1/4	1	1	2	3	2	3	1/2
O5	1/2	1/4	1/2	1/2	1	1	1/2	2	1/4
O6	1/4	1/9	1/4	1/3	1	1	1	1	1/7
O7	3	1/9	1/2	1/2	2	1	1	2	1/6
O8	1/6	1/9	1/5	1/3	1/2	1	1/2	1	1/9
O9	1	1/3	2	2	4	7	6	9	1

Attractive Quality(A)

Attractive Quality(A)	A1	A2	A3	A4	A5	A6
A1	1	5	1/5	1/2	1/3	1/7
A2	1/5	1	1/4	1/2	1/8	1/9
A3	5	4	1	5	1/3	1/3
A4	2	2	1/5	1	1/3	1/3
A5	3	8	3	3	1	1
A6	7	9	3	3	1	1

Expert 8:

Importance Matrix for First-Level Indicators:

Requirement Type	Must-have Quality(M)	One-dimensional Quality(O)	Attractive Quality(A)
Must-have Quality(M)	1	3	6
One-dimensional Quality(O)	1/3	1	2
Attractive Quality(A)	1/6	1/2	1

Matrix for Assessing the Importance of Secondary Indicators:

Must-have Quality(M)

Must-have Quality(M)	M1	M2	M3	M4
M1	1	5	9	9
M2	1/5	1	4	5
M3	1/9	1/4	1	3

M4	1/9	1/5	1/3	1

One-dimensional Quality(O)

One-dimensional Quality(O)	O1	O2	O3	O4	O5	O6	O7	O8	O9
O1	1	1/4	2	2	3	5	4	6	1/2
O2	4	1	4	4	7	8	1/2	9	1/2
O3	1/2	1/4	1	1	4	2	1	3	1/3
O4	1/2	1/4	1	1	2	1	1	2	1/3
O5	1/3	1/7	1/4	1/2	1	2	1/2	2	1/4
O6	1/5	1/8	1/2	1	1/2	1	1/2	3	1/7
O7	1/4	2	1	1	2	2	1	2	1/6
O8	1/6	1/9	1/3	1/2	1/2	1/3	1/2	1	1/9
O9	2	2	3	3	4	7	6	9	1

Attractive Quality(A)

Attractive Quality(A)	A1	A2	A3	A4	A5	A6
A1	1	2	1/4	1/2	1/3	1/4
A2	1/2	1	1/4	1/3	1/3	1/4
A3	4	4	1	1/2	1/2	1/3
A4	2	3	2	1	1/2	1/5
A5	3	3	2	2	1	1/3
A6	4	4	3	5	3	1

Expert 9:

Importance Matrix for First-Level Indicators:

Requirement Type	Must-have Quality(M)	One-dimensional Quality(O)	Attractive Quality(A)
Must-have Quality(M)	1	5	6
One-dimensional Quality(O)	1/5	1	2
Attractive Quality(A)	1/6	1/2	1

Matrix for Assessing the Importance of Secondary Indicators:

Must-have Quality(M)

Must-have	M1	M2	M3	M4

Quality(M)				
M1	1	5	9	7
M2	1/5	1	5	4
M3	1/9	1/5	1	1/3
M4	1/7	1/4	3	1

One-dimensional Quality(O)

One-dimensional Quality(O)	O1	O2	O3	O4	O5	O6	O7	O8	O9
O1	1	1/2	2	2	2	4	4	4	1/2
O2	2	1	5	3	8	8	9	9	1
O3	1/2	1/5	1	1	1	2	1/2	2	1/5
O4	1/2	1/3	1	1	2	4	3	4	1/3
O5	1/2	1/8	1	1/2	1	2	2	1/2	1/4
O6	1/4	1/8	1/2	1/4	1/2	1	1	1/5	1/8
O7	1/4	1/9	2	1/3	1/2	1	1	1	1/7
O8	1/4	1/9	1/2	1/4	2	5	1	1	1/9
O9	2	1	5	3	4	8	7	9	1

Attractive Quality(A)

Attractive Quality(A)	A1	A2	A3	A4	A5	A6
A1	1	2	1/6	1/4	1/7	1/8
A2	1/2	1	1/6	1/2	1/7	1/9
A3	6	6	1	2	1	1/2
A4	4	2	1/2	1	1/3	1
A5	7	7	1	3	1	1/4
A6	8	9	2	1	4	1

Expert 10:

Importance Matrix for First-Level Indicators:

Requirement Type	Must-have Quality(M)	One-dimensional Quality(O)	Attractive Quality(A)
Must-have Quality(M)	1	5	5
One-dimensional Quality(O)	1/5	1	2
Attractive Quality(A)	1/5	1/2	1

Matrix for Assessing the Importance of Secondary Indicators:

Must-have Quality(M)

Must-have Quality(M)	M1	M2	M3	M4
M1	1	4	8	9
M2	1/4	1	7	6
M3	1/8	1/7	1	2
M4	1/9	1/6	1/2	1

One-dimensional Quality(O)

One-dimensional Quality(O)	O1	O2	O3	O4	O5	O6	O7	O8	O9
O1	1	2	2	3	2	5	4	7	1/2
O2	1/2	1	1	1/3	5	9	9	9	1/2
O3	1/2	1	1	1	3	3	3	5	1/5
O4	1/3	3	1	1	2	2	2	3	1/3
O5	1/2	1/5	1/3	1/2	1	2	2	2	1/3
O6	1/5	1/9	1/3	1/2	1/2	1	1	1/2	1/7
O7	1/4	1/9	1/3	1/2	1/2	1	1	1/2	1/7
O8	1/7	1/9	1/5	1/3	1/2	2	2	1	1/8
O9	2	2	5	3	3	7	7	8	1

Attractive Quality(A)

Attractive Quality(A)	A1	A2	A3	A4	A5	A6
A1	1	1	1/4	1/2	1/4	1/3
A2	1	1	1/4	1/3	1/6	1/5
A3	4	4	1	1/2	1/3	1/4
A4	2	3	2	1	1/3	1/4
A5	4	6	3	3	1	1/3
A6	3	5	4	4	3	1

Expert 11:

Importance Matrix for First-Level Indicators:

Requirement Type	Must-have Quality(M)	One-dimensional Quality(O)	Attractive Quality(A)
Must-have Quality(M)	1	3	5
One-dimensional Quality(O)	1/3	1	3

Attractive Quality(A)	1/5	1/3	1

Matrix for Assessing the Importance of Secondary Indicators:

Must-have Quality(M)

Must-have Quality(M)	M1	M2	M3	M4
M1	1	4	9	9
M2	1/4	1	3	7
M3	1/9	1/3	1	4
M4	1/9	1/7	1/4	1

One-dimensional Quality(O)

One-dimensional Quality(O)	O1	O2	O3	O4	O5	O6	O7	O8	O9
O1	1	1/4	2	2	4	5	6	2	1/2
O2	4	1	3	3	5	9	9	9	1
O3	1/2	1/3	1	1	2	2	2	4	1/2
O4	1/2	1/3	1	1	1/2	3	1	3	1/3
O5	1/4	1/5	1/2	2	1	2	3	4	1/2
O6	1/5	1/9	1/2	1/3	1/2	1	1	2	1/6
O7	1/6	1/9	1/2	1	1/3	1	1	1/3	1/3
O8	1/2	1/9	1/4	1/3	1/4	1/2	3	1	1/4
O9	2	1	2	3	2	6	3	4	1

Attractive Quality(A)

Attractive Quality(A)	A1	A2	A3	A4	A5	A6
A1	1	2	1/5	1	1/4	1/3
A2	1/2	1	1/6	1/3	1/5	1/3
A3	5	6	1	1/2	2	2
A4	1	3	2	1	1/2	2
A5	4	5	1/2	2	1	2
A6	3	3	1/2	1/2	1/2	1

Expert 12:

Importance Matrix for First-Level Indicators:

Requirement Type	Must-have Quality(M)	One-dimensional	Attractive Quality(A)

		Quality(O)	
Must-have Quality(M)	1	3	9
One-dimensional Quality(O)	1/3	1	6
Attractive Quality(A)	1/9	1/6	1

Matrix for Assessing the Importance of Secondary Indicators:

Must-have Quality(M)

Must-have Quality(M)	M1	M2	M3	M4
M1	1	2	1	1/3
M2	1/2	1	1	1/2
M3	1	1	1	1/2
M4	3	2	2	1

One-dimensional Quality(O)

One-dimensional Quality(O)	O1	O2	O3	O4	O5	O6	O7	O8	O9
O1	1	1/2	2	2	3	5	4	6	1
O2	2	1	3	5	5	8	8	9	3
O3	1/2	1/3	1	1/2	3	2	1	1	1/3
O4	1/2	1/5	2	1	2	2	2	1/3	1/2
O5	1/3	1/5	1/3	1/2	1	2	2	2	1/4
O6	1/5	1/8	1/2	1/2	1/2	1	2	2	1/2
O7	1/4	1/8	1	1/2	1/2	1/2	1	2	1/5
O8	1/6	1/9	1	3	1/2	1/2	1/2	1	1/9
O9	1	1/3	3	2	4	2	5	9	1

Attractive Quality(A)

Attractive Quality(A)	A1	A2	A3	A4	A5	A6
A1	1	1/2	1/3	1/5	1/6	1/8
A2	2	1	1/4	1/5	1/9	1/9
A3	3	4	1	1	1/3	1/4
A4	5	5	1	1	1	1/3
A5	6	9	3	1	1	1/3
A6	8	9	4	3	3	1

Expert 13:

Importance Matrix for First-Level Indicators:

Requirement Type	Must-have Quality(M)	One-dimensional Quality(O)	Attractive Quality(A)
Must-have Quality(M)	1	2	5
One-dimensional Quality(O)	1/2	1	4
Attractive Quality(A)	1/5	1/4	1

Matrix for Assessing the Importance of Secondary Indicators:

Must-have Quality(M)

Must-have Quality(M)	M1	M2	M3	M4
M1	1	3	9	9
M2	1/3	1	7	8
M3	1/9	1/7	1	2
M4	1/9	1/8	1/2	1

One-dimensional Quality(O)

One-dimensional Quality(O)	O1	O2	O3	O4	O5	O6	O7	O8	O9
O1	1	1/5	2	1	2	4	4	7	1/3
O2	5	1	3	6	6	9	9	4	2
O3	1/2	1/3	1	1	4	2	5	3	1/3
O4	1	1/6	1	1	1	2	3	1/2	1/3
O5	1/2	1/6	1/4	1	1	2	1/2	2	1/3
O6	1/4	1/9	1/2	1/2	1/2	1	1/2	2	1/7
O7	1/4	1/9	1/5	1/3	2	2	1	2	1/7
O8	1/7	1/4	1/3	2	1/2	1/2	1/2	1	1/9
O9	3	1/2	3	3	3	7	7	9	1

Attractive Quality(A)

Attractive Quality(A)	A1	A2	A3	A4	A5	A6
A1	1	2	1/4	1/4	1/4	1/9
A2	1/2	1	1/3	1/2	1/3	1/9
A3	4	3	1	1/2	1	1/6
A4	4	2	2	1	1/3	1/6
A5	4	3	1	3	1	1/3

A6	9	9	6	6	3	1

Expert 14:

Importance Matrix for First-Level Indicators:

Requirement Type	Must-have Quality(M)	One-dimensional Quality(O)	Attractive Quality(A)
Must-have Quality(M)	1	2	8
One-dimensional Quality(O)	1/2	1	9
Attractive Quality(A)	1/8	1/9	1

Matrix for Assessing the Importance of Secondary Indicators:

Must-have Quality(M)

Must-have Quality(M)	M1	M2	M3	M4
M1	1	1	1	1/2
M2	1	1	3	1
M3	1	1/3	1	1
M4	2	1	1	1

One-dimensional Quality(O)

One-dimensional Quality(O)	O1	O2	O3	O4	O5	O6	O7	O8	O9
O1	1	1/4	2	1	1/4	4	4	2	1/2
O2	4	1	3	2	5	2	9	9	1/2
O3	1/2	1/3	1	1	2	3	3	3	1/3
O4	1	1/2	1	1	1/2	2	2	2	1/4
O5	4	1/5	1/2	2	1	1	2	2	1/4
O6	1/4	1/2	1/3	1/2	1	1	2	1	1/7
O7	1/4	1/9	1/3	1/2	1/2	1/2	1	1/2	1/3
O8	1/2	1/9	1/3	1/2	1/2	1	2	1	1/9
O9	3	1/2	3	3	3	7	7	9	1

Attractive Quality(A)

Attractive Quality(A)	A1	A2	A3	A4	A5	A6
A1	1	4	1/2	1/2	1/6	1/9
A2	1/4	1	1/4	1/3	1/6	1/5

A3	2	4	1	1	1/2	1/4
A4	2	3	1	1	1/3	1/2
A5	6	6	2	3	1	1
A6	9	5	4	2	1	1

Expert 15:

Importance Matrix for First-Level Indicators:

Requirement Type	Must-have Quality(M)	One-dimensional Quality(O)	Attractive Quality(A)
Must-have Quality(M)	1	3	9
One-dimensional Quality(O)	1/3	1	5
Attractive Quality(A)	1/9	1/5	1

Matrix for Assessing the Importance of Secondary Indicators:

Must-have Quality(M)

Must-have Quality(M)	M1	M2	M3	M4
M1	1	9	2	5
M2	1/9	1	1/6	1/6
M3	1/2	6	1	4
M4	1/5	6	1/4	1

One-dimensional Quality(O)

One-dimensional Quality(O)	O1	O2	O3	O4	O5	O6	O7	O8	O9
O1	1	1/4	2	1/2	1	1/2	1	6	1/2
O2	4	1	4	3	4	8	9	2	1/2
O3	1/2	1/4	1	1	2	2	3	5	1/4
O4	2	1/3	1	1	2	2	2	4	1/3
O5	1	1/4	1/2	1/2	1	2	1	2	1/2
O6	2	1/8	1/2	1/2	1/2	1	1	2	1/6
O7	1	1/9	1/3	1/2	1	1	1	1	1/7
O8	1/6	1/2	1/5	1/4	1/2	1/2	1	1	1/9
O9	2	2	4	3	2	6	7	9	1

Attractive Quality(A)

Attractive Quality(A)	A1	A2	A3	A4	A5	A6
A1	1	3	1/2	1/2	1/8	1/7
A2	1/3	1	1/5	1/2	1/7	1/9
A3	2	5	1	2	1/3	1/3
A4	2	2	1/2	1	1	1/6
A5	8	7	3	1	1	1/2
A6	7	9	3	6	2	1
